# Breaking Boundaries: Advancing Trisulfur Radical-Mediated Catalysis for High-Performance Lithium–Sulfur Batteries

**DOI:** 10.1007/s40820-025-01710-7

**Published:** 2025-04-11

**Authors:** Junfeng Wu, Bohai Zhang, Zhiqi Zhao, Yuehui Hou, Yufeng Wang, Ruizheng Zhao, Hao Zhang, Jiandong Hu, Ke Yang, Bin Tang, Zhen Zhou

**Affiliations:** 1 Henan International Joint Laboratory of Laser Technology in Agriculture Sciences, College of Mechanical and Electrical Engineering, Henan Agricultural University, Zhengzhou, 450002 People’s Republic of China; 2 Flavors and Fragrance Engineering and Technology Research Center of Henan Province, College of Tobacco Science, Henan Agricultural University, Zhengzhou, 450002 People’s Republic of China; 3 Interdisciplinary Research Center for Sustainable Energy Science and Engineering, School of Chemical Engineering, Zhengzhou University, Zhengzhou, 450001 People’s Republic of China; 4 Key Laboratory of Advanced Energy Materials Chemistry (Ministry of Education), Renewable Energy Conversion and Storage Center (ReCast), Nankai University, Tianjin, 300350 People’s Republic of China

**Keywords:** Lithium–sulfur batteries, Trisulfur radicals, Mediated catalyst, High donor number, Homogeneous/heterogeneous catalyst

## Abstract

Lithium–sulfur batteries (LSBs) have attracted significant attention due to their high theoretical energy density and low-cost raw materials. However, LSBs still face various challenges in practical applications, particularly the shuttle effect, electrode passivation, and slow kinetics. In recent years, trisulfur radicals (TRs), important intermediates in LSBs, have emerged as a promising and beyond-traditional solution to these problems, which serves as a mediated catalyst to improve the electrochemical performance of LSBs. As a system that is inconsistent with the catalytic conversion process discussed in the traditional LSBs, this review focuses on the generation, detection, promotion, and catalytic roles of TRs, especially emphasizing the formation of TRs in solid-state lapis lazuli analogs and discussing the pros and cons of high donor number solvents and/or their co-solvents in stabilizing TRs. Strategies involving homogeneous/heterogeneous catalysts are discussed for increment of TRs and enhancing catalytic reactions in LSBs. Ultimately, given TRs’ significant potential as a key factor in enhancing the performance of LSBs, future perspectives and outlooks are provided to guide the further development of TRs in LSBs. This review provides valuable insights into the design of electrolytes and catalysts for increment of TRs, paving the new practical direction and way for advanced LSBs.

## Highlights

- The review emphasizes the formation of trisulfur radicals in solid-state lapis lazuli analogs and the role of high donor number solvents and/or their co-solvents in stabilizing trisulfur radicals.
- The detection techniques are also discussed for monitoring the generation of trisulfur radicals, which are critical for understanding their behavior and optimizing the design of lithium–sulfur batteries.
- The strategies involving both homogeneous and heterogeneous catalysts are summarized to increase the generation of trisulfur radicals and enhance catalytic reactions in lithium–sulfur batteries for practical applications.

## Graphical abstract

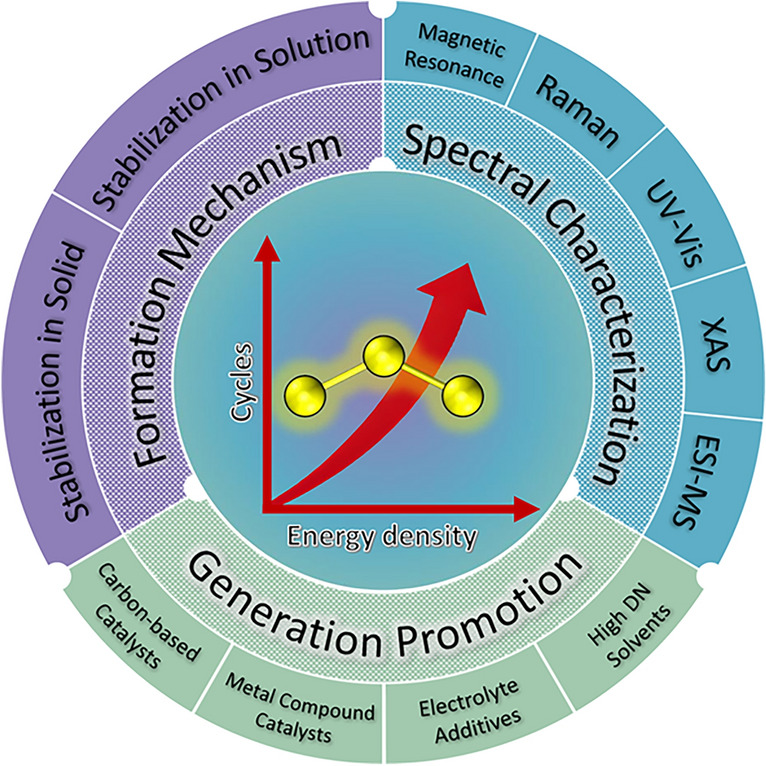

## Introduction

Typical lithium–sulfur batteries (LSBs), consisted with sulfur cathode and metallic lithium (Li) anode, are one of the most promising energy storage devices [1–3]. As a classical cathodic active material, elemental sulfur (*i.e.*, cyclo-S_8_) is natural abundant, cost-effective, and non-toxic. Meanwhile, it possesses a high theoretical specific capacity of 1675 mAh g^–1^ [4]. Metallic Li is the lightest metal (6.941 g mol^–1^), which possesses the highest specific capacity (3861 mAh g^–1^) among the metal electrodes. Moreover, Li has a conspicuous negative electrode potential (– 3.04 V *vs*. standard hydrogen electrode) [5–7]. Coupling cyclo-S_8_ and Li, the theoretical energy density of LSBs can reach 2600 Wh kg^–1^ or 2800 Wh L^–1^, which is much higher than the state-of-the-art Li-ion batteries [8, 9].

The operation of LSBs relies on the sulfur reduction reaction (SRR) occurring on the surface of sulfur. This process involves the dissolution of intermediate lithium polysulfides (LiPSs, chemical formula: Li_2_Sₓ, x equals to 3–8) and the deposition of Li_2_S_2_/Li_2_S, collectively referred to as the dissolution–deposition process. During this process, the diffusion of soluble LiPSs (such as Li_2_S_8_, Li_2_S_6_, Li_2_S_4_) leads to the "shuttle effect", while the deposition of insulating Li_2_S_2_/Li_2_S can easily passivate the electrode. These issues result in performance degradation and reduced lifespan of LSBs, posing significant challenges to their development and application.

To overcome the problems mentioned above, many efforts have been utilized, such as cathode design [10–12], separator functionalization [13, 14], electrolyte solution modification (additives and redox mediators) [15–17], lithium engineering technologies (surface stabilization and alloying) [18, 19], and electrocatalysts [20] that have been widely used in sulfur cathode. Rational design and use of electrocatalysts can accelerate the SRR process, reducing LiPSs accumulation and thereby mitigating the shuttle effect [21, 22]. Recently, multifunctional catalytic systems, such as triple-phase interface catalysis [23], selective catalysis [24], and hierarchical adsorption catalysis [25], have been proposed. These systems enable electrocatalysts to exhibit excellent adsorption and catalytic performance for LiPSs and simultaneously regulate the deposition of Li_2_S_2_/Li_2_S. Therefore, developing novel and multifunctional electrocatalysts represents a key approach to address the challenges faced by LSBs.

The choice of suitable electrolytes for LSBs is also caught in a dilemma, because the electrolytes not only serve as ion conductor for mass transport but also participate in the conversion reactions of LiPSs. The traditional electrolyte for LSBs is ether-based solution, *i.e.*, 1 M lithium bis(trifluoromethanesulfonyl)imide (LiTFSI) and 0.2 M LiNO_3_ in equivolume 1,3-dioxolane (DOL) and 1,2-dimethoxyethane (DME) [26, 27], noted as DOL/DME in this review. Soluble LiPSs are moderately dissolved to constitute a part of the catholyte. It is notable that minimizing amount of the electrolyte solution is essential for pursuing high energy density of LSBs [28, 29]. The ideal goal of practical LSBs in energy density is to achieve 500 Wh kg^–1^ or 700 Wh L^–1^, *viz.* a low electrolyte solution/sulfur (E/S) ratio of *ca.* 1 μL mg^–1^ is needed [30]. However, the E/S ratio of DOL/DME is limited to *ca.* 4.7 μL mg^–1^, which is far exceed the standard [31].

To reduce the amount of electrolyte or enhance the solubility of polysulfides, one effective approach is to use highly soluble electrolytes (HSEs) [32, 33]. For example, dimethyl sulfoxide (DMSO) that is the first investigated HSE for Li–S flow batteries has higher solubility for both long- and short-order polysulfides than DOL/DME [34]. The saturation concentration of Li_2_S_8_ exceeds 14 M [S] in pure DMSO, where it is higher than the required threshold of 10.4 M [S]. HSEs typically exhibit a higher donor number (DN). This value is defined as the negative enthalpy change associated with the formation of a 1:1 adduct between a Lewis base and the standard Lewis acid antimony pentachloride (SbCl_5_), in dilute solution in the non-coordinating solvent 1,2-dichloroethane with a zero DN [35, 36].

In essence, DN serves as a measure of a solvent's capacity to solvate cations and Lewis acids. For example, the DN of highly soluble DMSO is 29.8 kcal mol^–1^, whereas the DN of lowly soluble DME is 7.2 kcal mol^–1^ [35]. Thus, DN can be regarded as a descriptor for dissolving ability of LiPSs [37]. Long-order LiPSs, such as Li_2_S_6_ in a high-DN solvent, exhibits a blue coloration, which is attributed to the formation of trisulfur radicals or thiozonide anion $\left( {{\text{S}}_{3}^{ \bullet - } } \right)$ [38]. This anion is analogous to the ozonide anion $\left( {{\text{O}}_{3}^{ \bullet - } } \right)$, to which they are valence-isoelectronic [39]. Generally, the widely accepted formation mechanism of ${\text{S}}_{3}^{ \bullet - }$ radicals in high-DN solvents involves its derivation from the homolytic cleavage of the middle S–S bond in ${\text{S}}_{6}^{2 - }$ [40, 41].

The characterized absorption band (*ca.* 617 nm) of ${\text{S}}_{3}^{ \bullet - }$ radicals in high-DN solvents, such as DMSO, *N*,*N*-dimethylformamide (DMF), dimethylacetamide (DMA), or their mixture with low-DN solvent, can be easily distinguished in ultraviolet–visible (UV–Vis) spectrum [42, 43]. In other cases, ${\text{S}}_{3}^{ \bullet - }$ radicals can also be detected in DOL/DME mixtures with high-DN anions [43], or in low-DN tetraethylene glycol dimethyl ether (TEGDME or G4) solvents with more oxygen atoms available for coordination (4 per molecule) [44].

${\text{S}}_{3}^{ \bullet - }$ radicals serve as a key intermediate to enhance active sulfur interconversion reactions. First of all, the short-order Li_2_S_2_ or Li_2_S can react with the remanent cyclo-S_8_ or ${\text{S}}_{8}^{2 - }$ to form highly reactive ${\text{S}}_{3}^{ \bullet - }$ radicals during discharge, driving the full sulfur utilization of LSBs_._ What’s more, sulfur chemistry mediated by ${\text{S}}_{3}^{ \bullet - }$ radicals enables three-dimensional deposition of Li_2_S, which can weaken the surface passivation process, accelerating the electrode reaction kinetics [35, 42, 45]. In addition, ${\text{S}}_{3}^{ \bullet - }$ radicals additionally reduces the overpotential associated with Li_2_S oxidation during the charging process by providing extra oxidation reaction pathways [45]. However, high-DN solvents easily corrode the metallic Li anode, resulting in poor cycle performance and shortening cycle life of LSBs, which partly limits their further application [32].

There is another form of trisulfur radicals, namely lithium trisulfur radicals $\left( {{\text{LiS}}_{3}^{ \bullet } } \right)$, existing with similar effect of ${\text{S}}_{3}^{ \bullet - }$ radicals, which is derived from the homolytic cleavage of Li_2_S_6_ in the traditional and low-DN ether-based electrolyte [46]. ${\text{LiS}}_{3}^{\bullet}$ radicals can be regarded as an endogenous multifunctional electrocatalyst in traditional ether-based electrolyte system. Under the mediation/catalysis of ${\text{LiS}}_{3}^{\bullet}$ radicals, chemical reactions and electrochemical reactions in LSBs drive each other forward [40]. However, due to thermodynamic reasons, ${\text{LiS}}_{3}^{\bullet}$ radicals in traditional ether-based electrolytes are easily associated to form Li_2_S_6_ or undergo disproportionation reactions to form other sulfur species, leading to a small content. This is also the reason why ${\text{LiS}}_{3}^{\bullet}$ radicals difficultly be detected or distinguished by *ex situ* and steady-state testing techniques. For this reason, radicals trapping or spin trapping agents are used as a compromise method to stabilize ${\text{S}}_{3}^{\bullet-}$/${\text{LiS}}_{3}^{\bullet}$ radicals by forming radical adducts [47]. Otherwise, the transient generation of ${\text{LiS}}_{3}^{\bullet}$ radicals during the operation of LSBs with traditional low-DN ether-based electrolytes can be effectively detected, owing to the advanced *in situ* techniques (such as Raman spectroscopy [47], electron spin resonance (ESR) [48], X-ray absorption spectroscopy (XAS) [45], and UV–Vis [49]).

Due to the low content compared to total and the evanescent properties, ${\text{LiS}}_{3}^{\bullet}$ radicals are usually considered insufficient to affect the entire sulfur chemical reaction [50]. Nevertheless, a majority of as-reported catalysts, such as metal compounds, do not involve the mediation/catalysis of sulfur radicals, but focus more on their adsorption/catalysis [51, 52]. In recent years, some heterogeneous [53, 54] or homogeneous [55, 56] electrocatalysts were found to have the ability to induce the homolytic reaction of Li_2_S_6_ to generate more ${\text{LiS}}_{3}^{\bullet}$ radicals in the traditional ether-based electrolyte. At the same time, born with the rapid development of *in situ* technology and the improvement of the detection limit, ${\text{LiS}}_{3}^{\bullet}$ radicals were found to change significantly during the catalytic process. It can be speculated that sulfur radicals are likely to be one of the important factors for the catalytic effect of electrocatalysts in LSBs. Therefore, in-depth exploration of the incremental and efficiency improvement strategies and paths of ${\text{S}}_{3}^{\bullet-}$/${\text{LiS}}_{3}^{\bullet}$ radicals in LSBs are of great significance to the essential improvement of the performance of electrocatalysts in LSBs.

This review systematically summarizes the latest progress on ${\text{S}}_{3}^{\bullet-}$/${\text{LiS}}_{3}^{\bullet}$ radicals, focusing on their formation mechanisms in solid matter and solutions, spectral characterization techniques. The role of high-DN solvents and their co-solvent strategies, electrolyte additive strategies, and metal compound and carbon-based catalysts are proposed in promoting ${\text{S}}_{3}^{\bullet-}$/${\text{LiS}}_{3}^{\bullet}$ radicals generation. By comprehensively addressing the formation mechanisms, physicochemical properties, and catalytic behaviors of ${\text{S}}_{3}^{\bullet-}$/${\text{LiS}}_{3}^{\bullet}$ radicals, this review aims to provide new insights for the design of more efficient electrolytes and electrocatalysts. This effort not only deepens the understanding of the core reaction mechanisms, but also offers theoretical support for developing innovative strategies to improve energy density, rate capability, cycling stability, and overall performance of LSBs.

## Formation Mechanism of ${\mathbf{S}}_{\mathbf{3}}^{\bullet\mathbf{-}}$/${\mathbf{L}\mathbf{i}\mathbf{S}}_{\mathbf{3}}^{\bullet}$ Radicals

### Stabilization Mechanism in Solid Matter

**Fig. 1:**
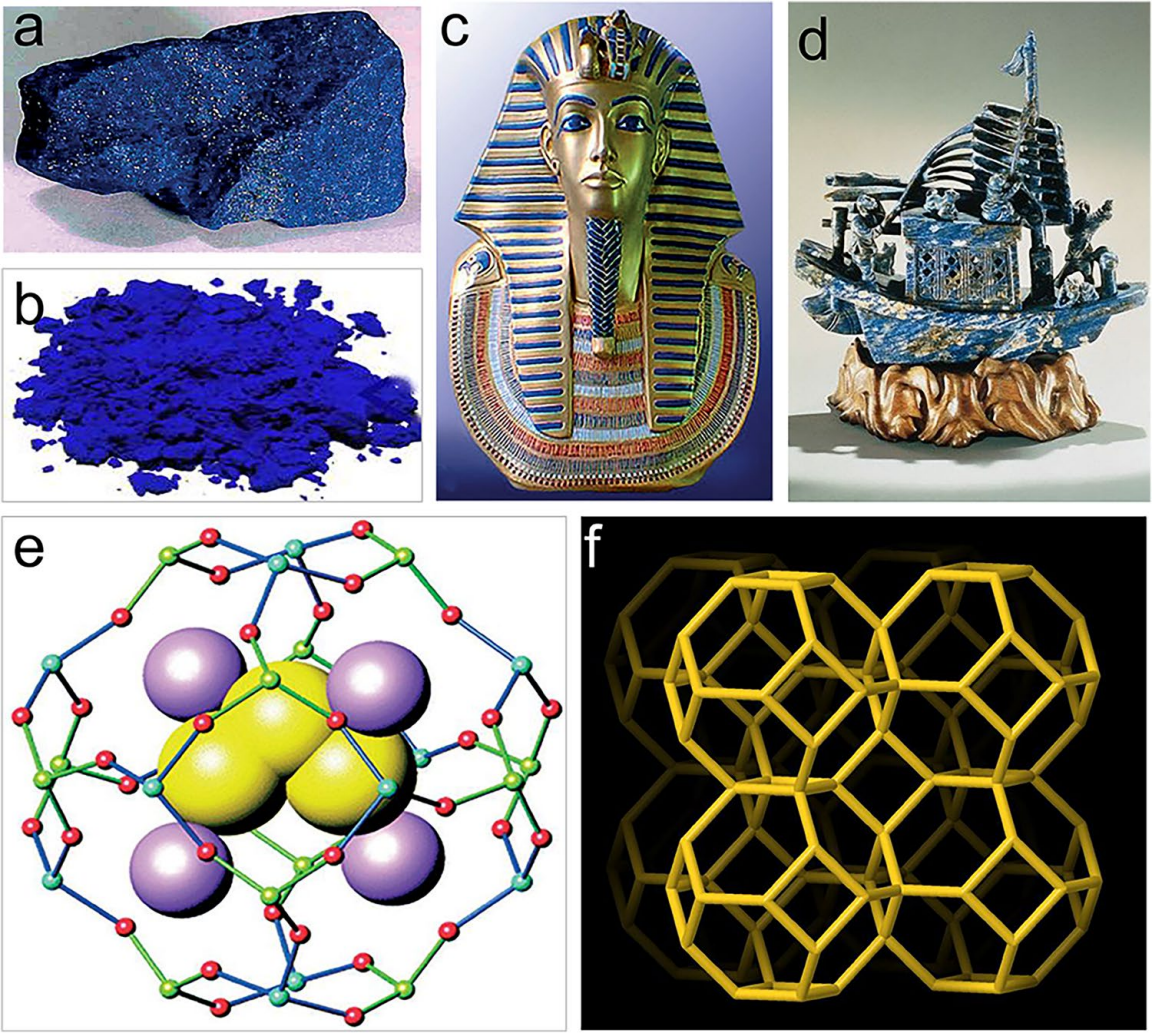
${\text{S}}_{3}^{\bullet-}$/${\text{LiS}}_{3}^{\bullet}$ radicals stabilized in solid matter. **a** Natural Lazurite mineral [57]. Copyright 2009, Elsevier. **b** The ultramarine pigments made from Lazurite [59]. Copyright 2018, American Chemical Society. **c** Gold mask of Pharaoh Tutankhamun with blue stripes [57]. Copyright 2009, Elsevier. **d** A craft of Chinese junk with a blue surface [61]. Copyright 1999, Royal Society of Chemistry. **e** Model of *β*-cage with assembled ${\text{S}}_{3}^{\bullet-}$ radical [57]. Copyright 2009, Elsevier. **f** SOD-type zeolite composed of *β*-cage [62]

Before the advent of modern chemical pigments, natural mineral Lazurite (Fig. 1a) [57], named from the Persian lajvard for blue [58], was always used to prepare an expensive blue pigment (Fig. 1b) [59] and exuded huge attraction to humans. Lazurite was concluded in a well-documented work that it originated from the mines in Badakhshan Province, Afghanistan [60]. It has been mined as a gem or pigment for more than 9000 years and was used as a pigment in painting and crafts since at least the sixth to seventh century. The blue stripe of famous gold mask of Pharaoh Tutankhamun in ancient Egypt (Fig. 1c) [57] and a craft of Chinese junk (Fig. 1d) [61] all use blue pigment coming from Lazurite mineral, showing high artistic value and attractiveness.

To date, we have known that natural blue pigment is a kind of lapis lazuli analogue [40, 63, 64]. Modern technology has confirmed that the blue color of natural pigments is caused by the replacement of some Na^+^ and Cl^–^ on the sodalite (SOD) cage or *β*-cage of the natural aluminosilicate sodalite (Na_8_[Al_6_Si_6_O_24_]Cl_2_) by some ${\text{S}}_{3}^{\bullet-}$ radicals to become Na_7_[Al_6_Si_6_O_24_]S_3_ (Fig. 1e) [57]. The ${\text{S}}_{3}^{\bullet-}$ radicals is located in the *β*-cage of lapis lazuli and is coordinated by seven Na^+^ in the center of the surrounding six-membered ring. It is expensive to extract lapis lazuli blue pigment from natural lapis lazuli minerals, but modern mature synthetic processes using zeolite as the main raw material can easily obtain lapis lazuli analogs. Zeolites are composed of [SiO_4_] and [AlO_4_]^−^ basic units, connected by Si–O-Al topology, and have hollow cages such as *α-*, *β-*, or *γ-*cage [65]. However, zeolites that can be used to synthesize lapis lazuli analogs are mainly sodalite-like type (Fig. 1f) [62], such as zeolite A (LTA), zeolite X/Y (FAU), zeolite EMT, and zeolite LTN, which are formed by connecting *β*-cages in different ways. Kowalak et al*.* mixed zeolite A with alkali metals and synthesized lapis lazuli analogs embedded with ${\text{S}}_{3}^{\bullet-}$ radicals at high temperature. Their results confirmed that calcination temperature and the type of mixed alkali metal cations (such as Li^+^, Na^+^, K^+^) affect the product type and yield [66]. Rejmak *et al.* conducted density functional theory (DFT) calculations on the structural properties of lapis lazuli analogs earlier, providing a reference for the analysis of related experimental spectral data [59].

Unlike zeolites found in nature, silicoaluminophosphate (SAPO) zeolites are synthesized artificially on a laboratory or industrial scale by replacing some of the Si and Al atoms in zeolite molecular sieves with P atoms. SAPO exhibits unique acidity, pore structures, and tunable properties. By ion exchange in the pores or on the surface of SAPO, Zn^+^ replaces the original cations (such as Na^+^, H^+^), resulting in Zn@SAPO, which alters the catalytic properties of SAPO and imparts unique acidity and active centers to the material. In terms of applications, Chen and co-workers demonstrated that the reaction of sublimed sulfur with Zn@SAPO-CHA produces a lapis lazuli analogue containing ${\text{S}}_{3}^{\bullet-}$ radicals, *i.e.*, (S_3_, Zn)@SAPO-CHA [64]. Due to electron transfer between the ${\text{S}}_{3}^{\bullet-}$ radicals and H_2_O molecules, this material can act as a sensor detecting trace amounts of H_2_O in air and organic solvents. This property where ${\text{S}}_{3}^{\bullet-}$ radicals can be accessible and interact with guest molecules offers valuable insights for using lapis lazuli analogues as ${\text{S}}_{3}^{\bullet-}$ radical donor electrocatalysts in LSBs.

Recent advancements in solid-state LSBs have shown great promise in improving battery performance by addressing issues such as dendrite growth and polysulfide dissolution. However, solid-state LSBs still face challenges such as low ionic conductivity and sluggish solid–solid sulfur redox reaction (SSSRR) [67–69]. ${\text{S}}_{3}^{\bullet-}$ radicals, with their high electrochemical reactivity and stability in lapis lazuli analogs, present an innovative solution to these challenges, with the analogy of assembling functional guest into zeolite cages as solid-state electrolytes [70]. Notably, lapis lazuli analogs are stable in high temperatures and exhibit excellent corrosion resistance [66], which can adapt to the preparation process conditions of solid-state electrolytes. In addition, these radical-carrier materials also possess electrically insulated properties and good ion conductivity, making them meet some basic requirements for solid-state electrolytes [71]. The ionic conductivity of lapis lazuli analogs may be improved by recombination into solid-state electrolytes, and the introduction of ${\text{S}}_{3}^{\bullet-}$ radicals is expected to promote SSSRR kinetics and electrode-solid electrolyte interface activity [72], leading to better cycle stability and higher rate performance of solid-state LSBs.

### Stabilization Mechanism in Solutions

Besides solid Lazurites of lapis lazuli analogs, ${\text{S}}_{3}^{\bullet-}$ radicals have been shown to be stable in aqueous solution under a pressure of 0.5 GPa and are expected to exist naturally at depth in the Earth's crust where subduction or high-pressure metamorphism occurs [73]. This radical ion is probably important in movement of copper and gold in hydrothermal fluids [74] and further influences sulfide chemistry under these environments.

The discovery and study of ${\text{S}}_{3}^{\bullet-}$ radicals in liquid solutions can be traced back to the observation of blue and red phenomena in polysulfide solutions. The earliest studies showed that sulfur in certain solvents (such as DMSO and DMF) could form solutions with deep blue colors, which aroused great interest among chemists. Initial studies found that sulfur dissolved in these solvents exhibited a deep blue color at low concentrations, which shifted to deep red when the polysulfide solution approaches saturation. This phenomenon was further proved by the unique chemical characteristics of sulfur in these solvents. The color transformation was closely related to the polarity and nucleophilicity of the solvent and was attributed to the solvent's role as an electron pair donor. In addition, sulfur can also produce blue solutions when heated with H_2_O and a small amount of alkaline salt. At first, it was believed that the blue and red substances in the solvent should be caused by neutral particles, but later it was confirmed that the sulfur molecules formed free radicals with unpaired electrons through electrochemical reduction or other reactions. Therefore, these radicals could form stable complexes with solvent molecules, thus making the solution show different colors.

**Fig. 2:**
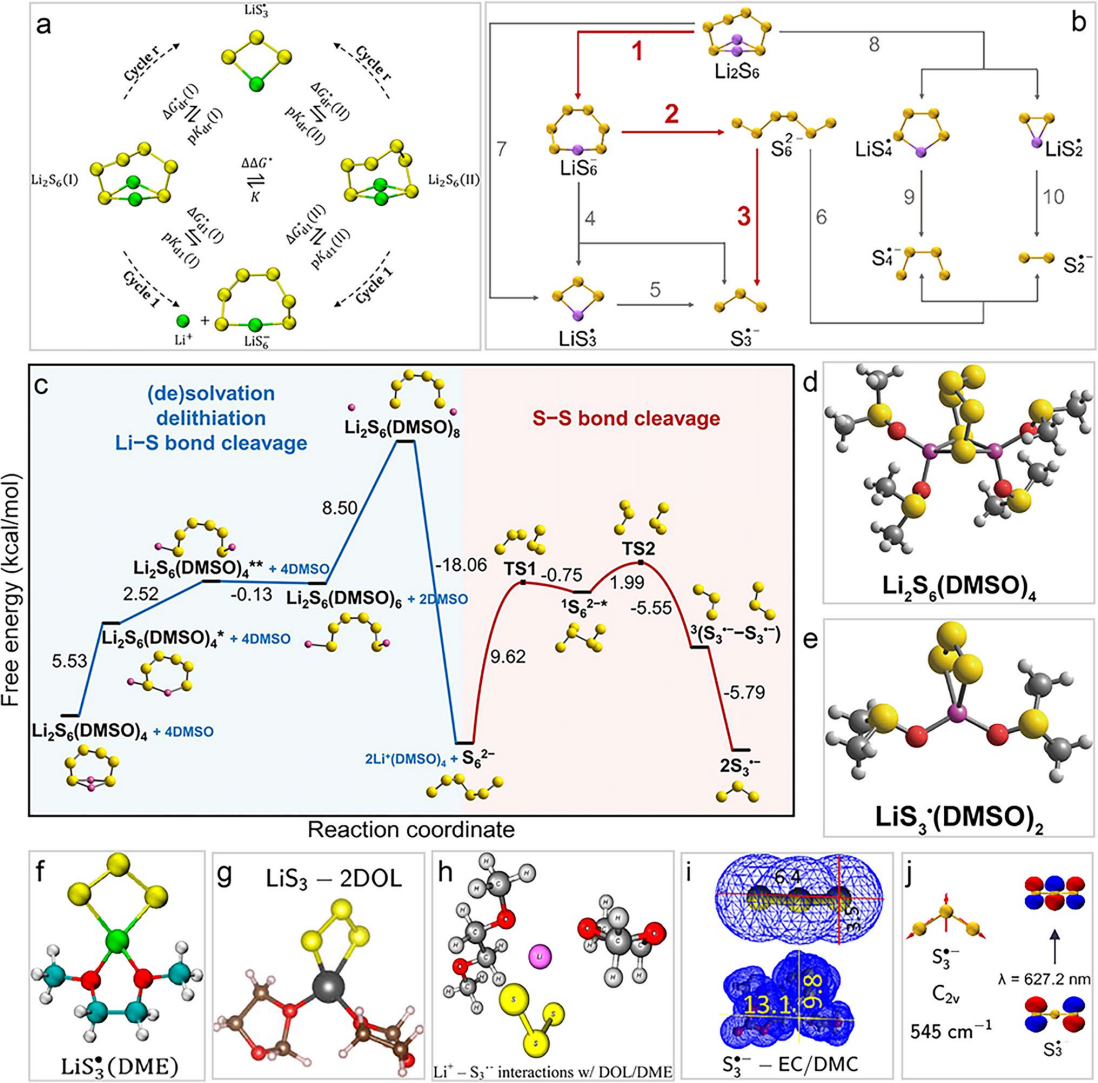
${\text{S}}_{3}^{\bullet-}$/${\text{LiS}}_{3}^{\bullet}$ radicals in solutions. **a** Chemical equilibria of polysulfides and ${\text{LiS}}_{3}^{\bullet}$ radicals in DME [46]. Copyright 2021, American Chemical Society. **b** Dissociation routes of Li_2_S_6_ in DMSO [79]. Copyright 2023, Royal Society of Chemistry. **c** Generation mechanism of ${\text{S}}_{3}^{\bullet-}$ radicals in DMSO [80]. Copyright 2023, Royal Society of Chemistry. **d** Optimized solvated structures of Li_2_S_6_(DMSO)_4_ cluster and **e** ${\text{LiS}}_{3}^{\bullet}$ (DMSO)_2_ cluster [80]. Copyright 2023, Royal Society of Chemistry. **f** Optimized gas-phase structures of Li ${\text{LiS}}_{3}^{\bullet}$ (DME) cluster [46], Copyright 2021, American Chemical Society. **g** ${\text{LiS}}_{3}^{\bullet}$(DOL)_2_ cluster [81], Copyright 2020, Springer Nature. **h** ${\text{LiS}}_{3}^{\bullet}$ (DOL-DME) cluster [82]. Copyright 2015, American Chemical Society. **i** Three-dimensional (3D) molecular models and Van der Waals surfaces of desolvated and dissolved ${\text{S}}_{3}^{\bullet-}$ radicals for the electrolyte system [83]. Copyright 2020, Springer Nature. **j** Normal mode analyses and electron transition model of ${\text{S}}_{3}^{\bullet-}$ [79]. Copyright 2023, Royal Society of Chemistry

It has been revealed that ${\text{S}}_{8}^{2-}$ will disproportionate into ${\text{S}}_{6}^{2-}$ and 1/4 S_8_ in electrolytes in LSBs [75–77]. Meanwhile, ${\text{S}}_{3}^{\bullet-}$ radicals can be also directly formed *via* the dissociation reaction of ${\text{S}}_{6}^{2-}\to 2{\text{S}}_{3}^{{\bullet}-}$, which is an entropy-driven process [41, 78]. It is worth noting that the dissociation pathways of LiPSs differ significantly between low-DN ether-based solvents (such as DME) and high-DN solvents (such as DMSO), which has been systematically studied by Zhang and co-workers [46, 79]. In low-DN solvents, Li_2_S_6_ primarily exists as a neutral molecule, with limited dissociation into Li^+^ and Li ${\text{S}}_{6}^{-}$ or homolysis into ${\text{LiS}}_{3}^{\bullet}$ radicals due to high dissociation constants (Fig. 2a) [46]. This results in a low concentration of radicals and minimal involvement in reaction pathways. In contrast, high-DN solvents, *e.g.,* DMSO, enhance the dissociation of Li_2_S_6_, leading to a significant formation of ${\text{S}}_{6}^{2-}$ and ${\text{S}}_{3}^{\bullet-}$ radicals (Fig. 2b) [79], stabilized by strong polarity.

These findings highlight the critical influence of solvent properties on the dissociation behavior of polysulfides and their impact on LSBs performance. Han et al*.* also proposed generation mechanism of ${\text{S}}_{3}^{\bullet-}$ radicals in DMSO (Fig. 2c) [80]. The ${\text{S}}_{6}^{2-}$ undergoes a series of dissociation and isomerization processes in high-DN solvents like DMSO. Initially, ${\text{S}}_{6}^{2-}$ forms clusters with DMSO, with intermediate species (such as Li_2_S_6_(DMSO)_4_, Li_2_S_6_(DMSO)_8_), following by releasing ${\text{S}}_{6}^{2-}$ and Li_2_S_6_(DMSO)_4_ with a free energy –18.06 kcal mol^–1^. Subsequently, ${\text{S}}_{6}^{2-}$ undergoes a singlet-state isomerization and transitions to a triplet state *via* a spin-state crossing, leading to the formation of ${\text{S}}_{3}^{\bullet-}$ radicals as form of ${\text{LiS}}_{3}^{\bullet}$(DMSO)_2_ with a kinetically feasible free energy barrier 9.62 kcal mol^–1^. The ${\text{S}}_{3}^{\bullet-}$ radical becomes somewhat shielded inside the caged Li^+^(DMSO)_2_ complex, which probably cannot easily access other solvated Li^+^ and result in longer lifetime and solubility. The configuration of ${\text{LiS}}_{3}^{\bullet}$(DMSO)_2_ is a natural analogy to superoxide radicals (${\text{O}}_{2}^{{\bullet}-}$) in the non-aqueous Li-air battery [84].

No matter Li_2_S_6_ and ${\text{LiS}}_{3}^{\bullet}$, Li^+^ predominantly adopts a tetrahedral coordination mode in DMSO (Fig. 2d, e), with lithium atoms directly bonded to nearby oxygen or sulfur atoms, showcasing the remarkable stability of this four-coordinated structure. This coordination behavior is widely observed in Li^+^ solvation and LiPSs systems across other solvents or solvent mixtures, such as DMA, DME, DOL, and DOL/DME blends (Fig. 2f-h) [46, 81, 82], playing a critical role in ensuring the structural stability and offering novel and specific design of functional solutions in LSBs. In fact, in the ${\text{S}}_{3}^{\bullet-}$ radicals’ solution, the solvent molecules and ${\text{S}}_{3}^{\bullet-}$ radicals are competing for the coordination with Li^+^. The entire solvated structure should be considered as a whole.

The highly reactive ${\text{S}}_{3}^{\bullet-}$ together with its parent ${\text{S}}_{6}^{2-}$ generated in LSBs is likely involved in the shuttling phenomenon, contributing to parasitic reactions with the metallic Li anode during extended cycling. Hence, when using the pore restriction strategy of the sulfur cathode to confine ${\text{S}}_{3}^{\bullet-}$ radicals, the size of the solvated cluster needs to be considered. After all, the size of ${\text{S}}_{3}^{\bullet-}$ radical (maximum 6.4 Å) is only half that of the solvated cluster (maximum 13.1 Å), as shown in Fig. 2i [83]. According to the calculation of Zhang et al*.* [79], ${\text{S}}_{3}^{\bullet-}$ radicals exhibits A1 irreducible representation in C_2v_ symmetry and gives rise to specific Raman-active symmetric stretching vibration of the S–S bond, and electronic transitions of n → π^∗^ resulting in the absorption at visible range of UV–Vis spectra (Fig. 2j). The Raman peak at 545 cm^−1^ and UV–Vis absorption at 617 nm is proven to be related to the existence of ${\text{S}}_{3}^{\bullet-}$ radicals via calculation. The calculation together provides theoretical basis and complementary methods for experimental detecting and characterizing ${\text{S}}_{3}^{\bullet-}$ radicals, enabling a detailed understanding of its structure and reactivity in polysulfide systems.

**Fig. 3:**
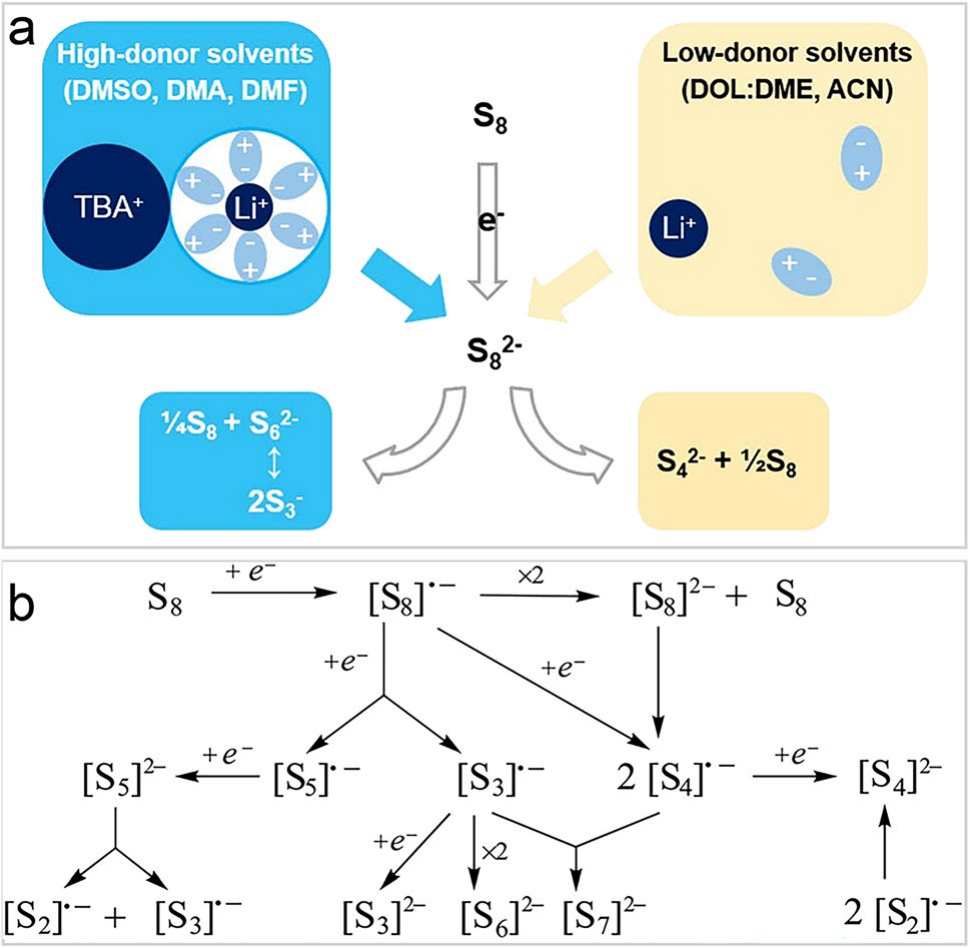
The formation mechanism of ${\text{S}}_{3}^{\bullet-}$ radicals. **a** Summary of how polysulfide stability/speciation is affected by its interaction with cations and solvent molecules [37]. Copyright 2018, IOP Publishing. **b** Possible radical anion intermediates in the stepwise electrochemical reduction of S_8_ [85]. Copyright 2023, Royal Society of Chemistry

To explore the stability of ${\text{S}}_{3}^{\bullet-}$ radicals in different solvents, Lu *et al.* used the hard and soft acids and bases (HSAB) theory to explain origin [37]. The ${\text{S}}_{4}^{2-}$ polysulfide (harder base) is better stabilized by weakly solvated Li^+^ (hard acid) prevalent in low-DN solvents, whereas ${\text{S}}_{3}^{\bullet-}$, ${\text{S}}_{8}^{2-}$, and ${\text{S}}_{6}^{2-}$ (softer bases) are better stabilized by strongly solvated Li^+^ (soft acid) prevalent in high-DN solvents (Fig. 3a) [37]. Among them, ${\text{S}}_{3}^{\bullet-}$, dissociated from ${\text{S}}_{6}^{2-}$, is identified as the softest base, which enables ${\text{S}}_{3}^{\bullet-}$ are more abundant in high-DN solvents. By contrast, owing to the stable properties of ${\text{S}}_{4}^{2-}$ in low-DN ether-based solvent, ${\text{LiS}}_{3}^{\bullet}$ radicals, dissociated from Li_2_S_6_ should be less.

The HSAB theory cannot explain all the issues occurring in LSBs. During the discharge process of LSBs, the concentration of ${\text{LiS}}_{3}^{\bullet}$ radicals reaches its maximum just before the onset of the plateau around 2.0 V. The dissociation of Li_2_S_6_ into ${\text{LiS}}_{3}^{\bullet}$ alone cannot fully explain this phenomenon. Park *et al.* proposed that the formation of ${\text{LiS}}_{3}^{\bullet}$ is also suggested to occur through electrochemical processes via Li + Li_2_S_6_ = Li_2_S_3_ + ${\text{LiS}}_{3}^{\bullet}$ [86]. This reaction pathway complements the dissociation model and underscores the importance of electrochemical conditions in driving the formation of reactive intermediates like ${\text{LiS}}_{3}^{\bullet}$ radicals, which contributes to the overall complexity of the polysulfide transformation process in LSBs.

In addition to ${\text{S}}_{3}^{\bullet-}$ radicals, other polysulfide radicals ${\text{S}}_{\text{n}}^{{\bullet}-}$ may also exist in the electrolyte [41, 85]. Their related generation paths are given in Fig. 3b. During the discharging process, the active sulfur (*viz.* cyclo-S_8_) on cathode gradually gains two electrons to form dianion (${\text{S}}_{8}^{2-}$) and dissolve in electrolyte [41]. Then, ${\text{S}}_{8}^{2-}$ will form various ${\text{S}}_{\text{n}}^{{\cdot}-}$ (n = 2–6) radicals that are known as potentially key intermediates in the electrochemical reduction of cyclo-S_8_ in LSBs [87]. Moreover, ${\text{S}}_{\text{n}}^{{\cdot}-}$ radicals obtain one more electron to form closed-shell dianions (such as ${\text{S}}_{6}^{2-}$ and ${\text{S}}_{3}^{2-}$) corresponding to them. ${\text{S}}_{\text{n}}^{{\cdot}-}$ may also form longer chain closed-shell dianions, *e.g.,* ${\text{S}}_{10}^{2-}$ and ${\text{S}}_{12}^{2-}$ via symmetric coupling of ${\text{S}}_{5}^{{\bullet}-}$ and ${\text{S}}_{6}^{{\bullet}-}$, respectively, while association of ${\text{S}}_{3}^{\bullet-}$ and ${\text{S}}_{4}^{{\bullet}-}$ yields ${\text{S}}_{7}^{2-}$ [88, 89]. Similarly, Prendergast and co-workers think that ${\text{LiS}}_{4}^{\bullet}$ and ${\text{LiS}}_{5}^{\bullet}$, generated from the homolytic and heterolytic reaction of Li_2_S_8_ via Li_2_S_8_ $\to$ 2 ${\text{LiS}}_{4}^{\bullet}$ and Li_2_S_8_ $\to$ ${\text{LiS}}_{3}^{\bullet}$ + ${\text{LiS}}_{5}^{\bullet}$, respectively, also exist, by fitting the experimental XAS spectrum with calculated ones [90]. Even more, Kawase *et al.* think other radicals, such as ${\text{LiS}}_{2}^{\bullet}$, ${\text{LiS}}_{6}^{\bullet}$, ${\text{LiS}}_{7}^{\bullet}$ and ${\text{LiS}}_{8}^{\bullet}$, exist through fitting experimental UV–Vis spectra with calculated ones [91]. However, there is no direct evidence of the existence of ${\text{S}}_{\text{n}}^{{\bullet}-}$ radicals, except for ${\text{S}}_{3}^{\bullet-}$ radicals, since the overlapping of spectra signals [92].

## Spectral Characterization Technique Detecting ${\mathbf{S}}_{\mathbf{3}}^{{\bullet}\mathbf{-}}$/${\mathbf{L}\mathbf{i}\mathbf{S}}_{\mathbf{3}}^{\bullet}$ Radicals

### Magnetic Resonance Spectroscopy

${\text{S}}_{3}^{\bullet-}$/${\text{LiS}}_{3}^{\bullet}$ radicals in LSBs have been fully characterized by a variety of spectroscopic techniques. Therein, ESR spectroscopy is a critical tool for identifying these paramagnetic radicals in solution and solid catalyst defects (such as oxygen vacancy and sulfur vacancy in metal oxides) with the spin transitions of unpaired electrons. This unpaired electron interacts with external static magnetic field and are motivated from lower to higher energy levels by low-energy microwave (on the order of meV) in ESR test. Besides, ESR tests are usually operated in dark and low-temperature condition. Thus, this applied microwave radiation has a small effect on the polysulfide ions, enabling ESR technique to have a strong anti-interference ability in detect ${\text{S}}_{3}^{\bullet-}$ radicals.

**Fig. 4:**
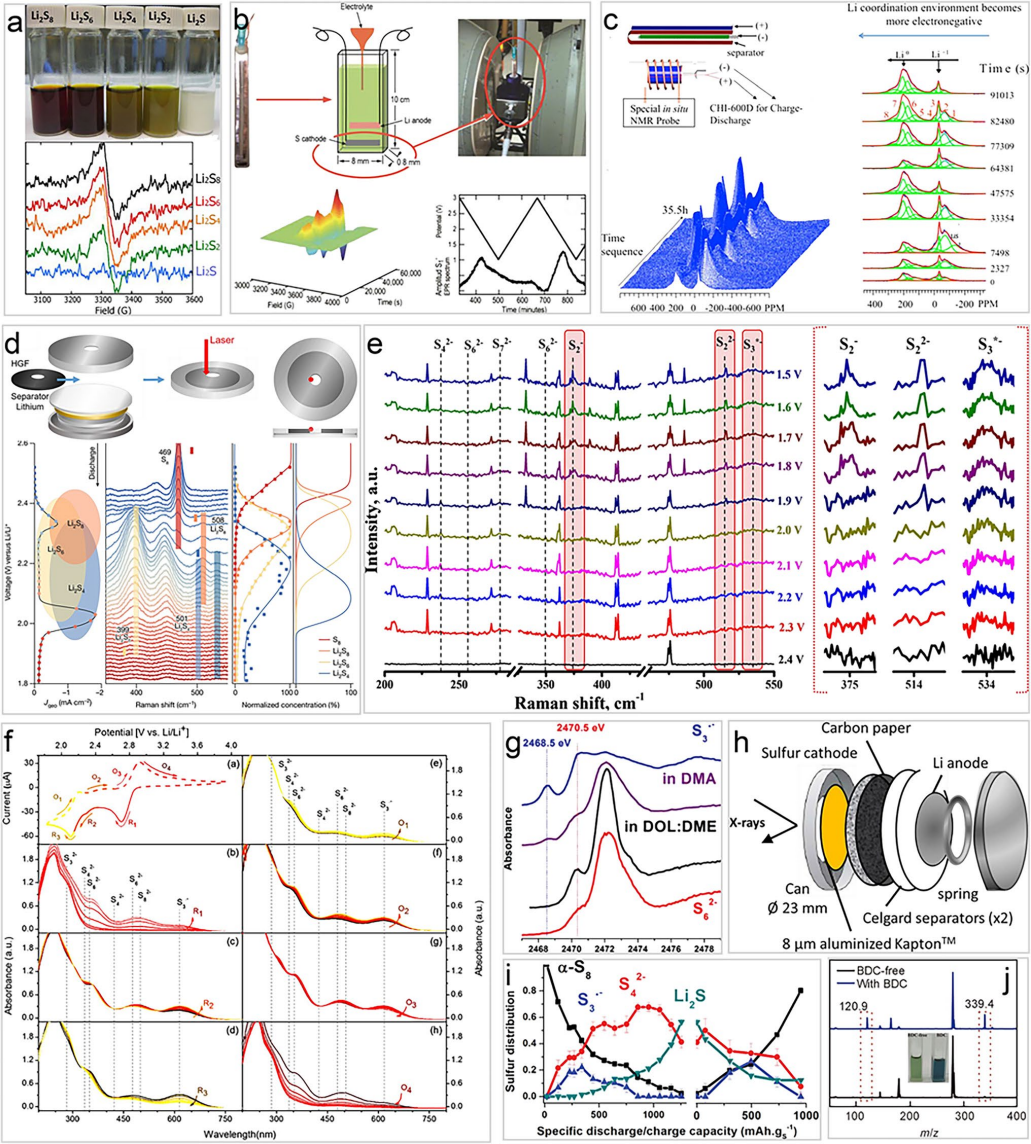
Spectrum technologies to detect ${\text{S}}_{3}^{\bullet-}$/${\text{LiS}}_{3}^{\bullet}$ radicals. **a** The upper: Li_2_S_x_ in DOL/DME solvents, and the bottom: corresponding EPR spectra [48]. Copyright 2015, IOP Publishing. **b** EPR spectra for *in situ* testing and capturing of radical resonance signals [48]. Copyright 2015, IOP Publishing. **c** *In situ* NMR probe and the acquired spectra [82]. Copyright 2015, American Chemical Society. **d** *In situ* Raman results with the carbon-based catalytic electrode and DOL/DME electrolyte [50]. Copyright 2024, Springer Nature. **e** *In situ* Raman measurements for LiPS redox species evolution on carbon-based cathode and TEGDME electrolyte [93]. Copyright 2022, IOP Publishing. **f** *In operando* UV − Vis spectra of each reaction step with carbon paper cathode and DMSO-based S_8_ catholyte [35]. Copyright 2016, American Chemical Society. **g** K-edge XANES spectra from discharging LSBs using the liquid electrolytes compared with the reference materials [45]. Copyright 2015, John Wiley and Sons. **h** Schematic of the operando cell for in situ XAS [94]. Copyright 2013, American Chemical Society. **i** Linear combination fit analysis of the XANES spectra upon cycling [45]. Copyright 2015, John Wiley and Sons. **j** ESI–MS results of BDC-free and BDC electrolyte solutions [49]. Copyright 2024, Springer Nature

Figure 4a shows the *ex situ* ESR of several prepared polysulfides solution with DOL/DME, revealing that ${\text{S}}_{3}^{\bullet-}$ radicals are present in most situation, due to the rapid, non-stepwise disproportionation of ${\text{S}}_{8}^{2-}$, ${\text{S}}_{6}^{2-}$, and ${\text{S}}_{4}^{2-}$. In view of the high precision of the detection of ${\text{S}}_{3}^{\bullet-}$ radicals by ESR spectrum, the weak changes of ${\text{S}}_{3}^{\bullet-}$ radicals during the operation of LSBs can be well captured by *in situ* ESR spectrum (Fig. 4b) [48]. Persistent during electrochemical cycling, the content of the ${\text{S}}_{3}^{\bullet-}$ radicals shows a significant change, indicating they actively participate in sulfur conversion reaction pathways, making their precise detection by ESR essential for understanding and optimizing polysulfide systems in LSBs.

In addition to directly detecting ${\text{S}}_{3}^{\bullet-}$ radicals, another magnetic resonance technology, nuclear magnetic resonance (NMR), based on the principle of interaction between nuclear spin and external magnetic field, is also used to indirectly detect the presence of ${\text{S}}_{3}^{\bullet-}$ radicals. As shown in Fig. 4c, the chemical shift of ^7^Li changes arises from the dynamic coordination environment of Li^+^ with different sulfur-based species, such as ${\text{S}}_{3}^{\bullet-}$ radicals, in polysulfide systems [82]. These chemical shifts that correspond to different lithium nuclei (approximately − 58 ± 10 ppm) do not overlap with the signal range of LiPSs (such as Li_2_S_*x*_, ± 2 ppm), but clearly distinguishing ${\text{S}}_{3}^{\bullet-}$ radical signals from polysulfide signals. Besides, in in situ environments, ${\text{S}}_{3}^{\bullet-}$ radical signals are particularly prominent due to the significant increase in radical concentration during electrochemical reactions, further enhanced by local electric fields and charged surfaces. The unique sensitivity and non-interference of *in situ* NMR provide a supplementary tool for studying ${\text{S}}_{3}^{\bullet-}$ radicals and their critical role in polysulfide reaction mechanisms of LSBs.

### Raman Spectroscopy

Raman spectroscopy is another tool based on photon scattering technology capable of detecting ${\text{S}}_{3}^{\bullet-}$ radicals with its characteristic vibrational peak at 531 cm^−1^ and other LiPSs, enabling the study of sulfur radical dynamics and their role in the polysulfide reaction mechanisms during the operation of LSBs. Liu *et al.* identify key intermediate species and their transformations by monitoring the characteristic vibrational peaks of polysulfides during discharge (Fig. 4d) [50]. For example, the initially detected S_8_ peak (469 cm^−1^) gradually disappears, while peaks corresponding to Li_2_S_8_ (508 cm^−1^), Li_2_S_6_ (399 cm^−1^), and Li_2_S_4_ (501 cm^−1^) emerge and reach their respective maxima, reflecting the electrochemical conversion and equilibrium reactions between polysulfides. Additionally, a minor Raman peak (531 cm^−1^) attributed to the ${\text{LiS}}_{3}^{\bullet}$ was detected, which is associated with a homolytic reaction of the electrochemically inactive Li_2_S_6_ (Li_2_S_6_ ↔ 2${\text{LiS}}_{3}^{\bullet}$).

Although the concentration of ${\text{LiS}}_{3}^{\bullet}$ radicals in the low-DN electrolyte, such as traditional ether-based electrolyte, is relatively low (less than 3% of Li_2_S_6_), ${\text{LiS}}_{3}^{\bullet}$ was thought to have limited impact on the equilibrium of the overall reaction network. However, because of the high sensitivity of Raman spectroscopy that enables the detection of these critical and transient species, this provides valuable insights and evidence into the mechanism revelation of radicals involvement in LSBs. Similarly, Thangavel *et al.* used TEGDME, a low-DN solvent, as the electrolyte solvent coupled with a carbon cloth electrode, under which the *in situ* Raman spectroscopy signal was detected only corresponding to weak ${\text{S}}_{3}^{\bullet-}$ radicals (Fig. 4e) [93]. Herein, Li_2_S_6_ is considered electrochemically inactive when using carbon electrodes, as described in this and the previous reference. However, when the electrode was replaced with metallic Ni, the ${\text{S}}_{3}^{\bullet-}$ radicals’ signal was significantly enhanced. This enhancement is likely due to the strong polar–polar interactions between the Ni electrode and Li_2_S_6_, *i.e.*, the Ni electrode adsorbs and stabilizes Li_2_S_6_. This phenomenon highlights the critical role of metal electrodes in promoting the formation of trisulfur radicals. Inspired by this, emerging single-atom catalysts (SACs), particularly those based on transition metals, have shown potential in enhancing the electrochemical reactions in LSBs [95]. SACs provide a high density of active sites with precise control over atomic coordination [96], which can effectively stabilize polysulfides, improve reaction kinetics, and facilitate the formation of trisulfur radicals. The unique properties of SACs could thus offer significant advantages in optimizing LSB performance. Similar phenomena will be discussed later in Sect. 4.4. The increment of Li_2_S_6_ leads to the dissociation of more ${\text{LiS}}_{3}^{\bullet}$ and altering the electrochemical reaction pathway. These results make Raman technology vital in detecting ${\text{S}}_{3}^{\bullet-}$/${\text{LiS}}_{3}^{\bullet}$ radicals of LSB system, but it need to consider the effect of light source (commonly used 532, 632.8 nm *etc*.) on polysulfide, which has been proved by Zhang and co-workers [79].

### UV–Vis Spectroscopy

UV–Vis spectroscopy is another effective method based on electron exciting for monitoring blue color ${\text{S}}_{3}^{\bullet-}$ radicals solution, differing from Raman spectroscopy through vibrational signatures. UV–Vis spectroscopy using ultraviolet and visible light sources (< 700 nm) identifies the electronic transitions signature of ${\text{S}}_{3}^{\bullet-}$ radicals, such as n → π^∗^, through its characteristic absorption peak at 617 nm, enabling dynamic, time-resolved tracking of ${\text{S}}_{3}^{\bullet-}$ radicals concentrations during LSBs operation. Its sensitivity to electronic environments allows selective detection of ${\text{S}}_{3}^{\bullet-}$ radicals even in complex polysulfide systems, providing valuable information on reaction kinetics.

Zou *et al.* used UV–Vis to track the evolution of polysulfide species with cyclic voltammetry (CV) operation, revealing key reduction and oxidation mechanisms (Fig. 4f) [35]. During reduction, cyclo-S_8_ undergoes stepwise transformations to ${\text{S}}_{8}^{2-}$, ${\text{S}}_{6}^{2-}$, ${\text{S}}_{4}^{2-}$, ${\text{S}}_{3}^{\bullet-}$, and ${\text{S}}_{3}^{2-}$ in DMSO. Each reaction shows distinct kinetics, with ${\text{S}}_{8}^{2-}$ reaching a steady state quickly, while ${\text{S}}_{6}^{2-}$ disproportionation (${\text{S}}_{8}^{2-}\to {\text{S}}_{6}^{2-}$ + $1/4$ S_8_ $\downarrow$) and ${\text{S}}_{3}^{{\bullet}-}$ radicals generation (${\text{S}}_{6}^{2-}\to 2{\text{S}}_{3}^{{\cdot}-}$ or 3 ${\text{S}}_{4}^{2-}\to 2{\text{S}}_{3}^{{\bullet}-}$+2 ${\text{S}}_{3}^{2-}$) take longer. During oxidation, polysulfides are reformed and ultimately converted back to cyclo-S_8_. This observation indicates that ${\text{S}}_{3}^{\bullet-}$ radicals are the most stable and dominant reaction intermediates in high-DN solvents. The study highlights the central role of ${\text{S}}_{3}^{\bullet-}$ radicals in redox processes (${\text{S}}_{3}^{\bullet-}$+ e^−^ $\to {\text{S}}_{3}^{2-}$) and demonstrates the value of UV–Vis spectroscopy in correlating electrochemical potential with the dynamic behavior of polysulfides. However, it may need to consider the photosensitive characteristic of the dissociation reaction of ${\text{S}}_{6}^{2-}$ into ${\text{S}}_{3}^{\bullet-}$ radicals when making a quantitative analysis [79].

### XAS Spectroscopy

XAS is a highly powerful analytical technique that provides information about the valence state, local structure, and coordination environment of elements in a sample. The high-energy X-ray (up to 10 keV) with 0.01–10 nm wavelength is enough to excite the inner electrons (such as K-shell or L-shell electrons) out of the atom, producing an X-ray absorption spectrum. Through XAS testing, researchers can gain deep insights into the electronic structure, chemical state and local geometry of the atom of trisulfur radicals and other polysulfides [90], including their coordination with catalysts, metal ions or solvent molecules. This coordination significantly influences their electrochemical behavior, reaction kinetics, and stability during cycling of in LSBs. By analyzing both the X-ray absorption near edge structure (XANES) and extended X-ray absorption fine structure (EXAFS) regions, the local atomic environment, oxidation states, and bond distances can be correlated with the reactivity and stability of sulfur species [45, 97]. Therefore, the ability of *in situ* XAS to track the evolution and relationship of various sulfur species is crucial for understanding structure–property relationship, offering valuable insights into how these features affect battery performance and help to design better sulfur cathodes and electrolytes.

Wujcik *et al*. simulated the XANES spectra of the ${\text{LiS}}_{3}^{\bullet}$ radicals dissolved in TEGDME, based on ab initio molecular dynamics (AIMD) sampling performed at 298 K [45]. This simulation shows that the terminal sulfur 1*s*−3*p* (π^∗^) transition of ${\text{LiS}}_{3}^{\bullet}$ radical give rise to the characteristic peak at 2468.5 eV, which can be easily distinguished from other polysulfide dianions. Since the physical state (crystalline, amorphous, or solute) does not affect the spectral features in the XANES region, the XAS spectrum of a ultramarine pigment of lapis lazuli analog containing ${\text{S}}_{3}^{\bullet-}$ radicals can be used as a reference spectrum for detecting ${\text{S}}_{3}^{\bullet-}$ radicals in solution (Fig. 4g). Experimental results show that there is a relatively weak and narrow peak (2468.5 eV) below the pre-edge peak (2470.5 eV) of polysulfide dianions in the reference spectrum, which is in accordance with theoretical calculation, thereby proving that the characteristic peak at 2468.5 eV belongs to the ${\text{S}}_{3}^{\bullet-}$ radical for the blue solution of polysulfides.

According to XAS experimental spectra, Cuisinier *et al.* further demonstrated that ${\text{S}}_{6}^{2-}$ readily dissociated into ${\text{S}}_{3}^{\bullet-}$ radicals in high-DN solvents, such as DMA and DMSO, resulting in high radical concentrations [45]. By using *in situ* XANES technology and the special electrode (Fig. 4h) [94], the content of ${\text{S}}_{3}^{\bullet-}$ varies with the discharge and charge process up to 25% of the total sulfur (Fig. 4i) [45]. Thus, its role as an internal redox mediator is confirmed, enabling 24% more sulfur utilization of DMA-based electrolyte than low-DN TEGDME electrolyte. Conversely, ${\text{S}}_{3}^{\bullet-}$ radicals are not stabilized in a measurable (*i.e.*, ≪ 5%) concentration in traditional ether-based electrolytes at the experimental timescale (*ca.* a few minutes). In addition, ${\text{S}}_{3}^{\bullet-}$ radicals are found to reacts with DOL at elevated temperatures, while DME remains inert. So, DOL as a co-solvent with high-DN solvent should take into account the effect of temperature. These findings highlight the critical role of ${\text{S}}_{3}^{\bullet-}$ radicals dynamics, solvent interactions, and the importance of anode protection for utilizing high-DN solvents in LSBs. There is no doubt that the higher energy of XAS than UV–Vis makes it difficult to quantitatively analyze ${\text{S}}_{3}^{\bullet-}$ radicals and polysulfides.

### Mass Spectrometry

${\text{S}}_{3}^{\bullet-}$ radicals are challenging to be detected in ether-based solvents due to their low stability and rapid conversion to other polysulfide species. However, biphenyl-4,4’-dicarboxylic acid (BDC) serves as a stabilizer, enhancing the formation and stability of ${\text{S}}_{3}^{\bullet-}$ radicals coordination compound through strong Lewis acid–base interactions. Dou *et al.* confirms that the presence of ${\text{S}}_{3}^{\bullet-}$ radicals becomes prominent in the presence of BDC using UV–Vis spectroscopy in the form of a strong characteristic absorption peak at 612 nm (upper panel of Fig. 4j) [49]. This stabilization of coordination compound also enables electrospray ionization-mass spectrometry (ESI–MS) to detect specific mass-to-charge ratios (m/z) corresponding to [BDC-${\text{S}}_{3}^{\bullet-}$] complexes at m/z = 339.4 kg C^−1^ (bottom panel of Fig. 4j), providing direct evidence of the radical and its interactions. Together, these findings highlight the critical role of BDC in facilitating the detection and study of ${\text{S}}_{3}^{\bullet-}$ radicals in systems where it would otherwise remain elusive.

## Promoting Strategies for the Generation of ${\mathbf{S}}_{\mathbf{3}}^{{\bullet}\mathbf{-}}$/${\mathbf{L}\mathbf{i}\mathbf{S}}_{\mathbf{3}}^{\bullet}$ Radicals

The unique electronic structure of sulfur radicals, including unpaired electrons, electron delocalization, and SOMO-LUMO energy level characteristics, gives them high electron transfer efficiency over sulfur in redox reactions. This makes sulfur radicals highly reactive in organic synthesis, promoting C–S bond formation, cyclization reactions, and sulfurization reactions, and they are widely used in the green synthesis of sulfur-containing compounds and heterocyclic molecules [98, 99]. Similarly, these characteristics make sulfur radicals key intermediates in LSBs, contributing to enhance sulfur utilization and reaction kinetics.

The ${\text{S}}_{3}^{\bullet-}$ radical is relatively stable and primarily exists in high-DN solvents such as DMSO, while the ${\text{LiS}}_{3}^{\bullet}$ radical is less stable and exists in lower concentrations in traditional low-DN solvents such as DOL/DME. Regarding reactivity, the ${\text{LiS}}_{3}^{\bullet}$ radical should have higher reactivity than ${\text{S}}_{3}^{\bullet-}$ due to the higher vertical electron affinity (1.9 eV) of ${\text{LiS}}_{3}^{\bullet}$ against the more negative value (~ –3.0 eV) of ${\text{S}}_{3}^{\bullet-}$ [79]. However, note that both of these radicals are highly reactive species in their respective electrolyte systems. The enhanced stability of ${\text{S}}_{3}^{\bullet-}$ in high-DN solvents contributes to improved sulfur utilization and reduced discharge/charge overpotential in LSBs compared with those using traditional low-DN solvents. On the other hand, the high reactivity of ${\text{LiS}}_{3}^{\bullet}$ radicals makes low-DN LSBs promising candidates, as these radicals can act as a mediating catalyst, due to better compatibility with the lithium anode than high-DN solvent systems. The following sections reviews strategies that promote the stability of reactive radicals, while minimizing the adverse effects of high-DN solvents, as summarized in Table 1.

**Table 1:** Comparison of parameters and performances of LSBs with different strategies for generation promotion of trisulfur radicals

Strategy	Catalyst/Material type	Initial capacity (mAh g^−1^)	E/S (μL mg^−1^)	Sulfur Loading (mg cm^−1^)	Rate(C)	Cycles	Dosage	References
High-DN solvent	DMSO	1250	~ 10.0	~ 6.0	–	–	50 v%	[42]
High-DN solvent	DMI^a^	1595	~ 5.0	10.0	0.1	~ 50	50 v%	[100]
Co-solvent	TMU^b^ /DOL	1524	3.0	2.5	0.1	180	50 v%	[43]
High-DN solvent additive	NMP^c^	1250	7.1	4.2	0.3	340	1 v%	[101]
High-DN anion additive	Br⁻	1535	–	~ 3.0	0.2	80	1 M	[102]
High-DN anion additive	FL^d^	1099	5.0	5.5	0.5	520	50 mM	[103]
High-DN anion additive	DPDTe^e^	1227	5.0	5.0	1.0	300	50 mM	[104]
Metal compound catalyst	WO_3−*x*_	1221	20.0	2.5	4.0	300	4.5 wt%^f^	[53]
Metal compound catalyst	VS_2−*x*_	1471	15.0	5.6	0.2	400	10 wt%^f^	[105]
Metal compound catalyst	Co_9_S_8_/MoS_2_-rGO	1129	13.8	3.2	0.5	300	30 wt%^f^	[106]
MXene	Ti_3_C_2_T_*x*_	~ 1000	8.0	10.5	–	80	36 wt%^f^	[107]
Carbon-based catalyst	UN/O-CNS	1211	–	4.2	1.0	1500	32 wt%^f^	[54]

^a^DMI refers to 1,3-dimethyl-2-imidazolidinone. ^b^TMU refers to tetramethylurea. ^c^NMP refers to N-methyl-2-pyrrolidone. ^d^FL refers to fluorenone. ^e^DPDTe refers to diphenyl ditelluride. ^f^Calculated based on the overall mass of the cathode composite

### High-DN Solvents and Their Co-Solvents

**Fig. 5:**
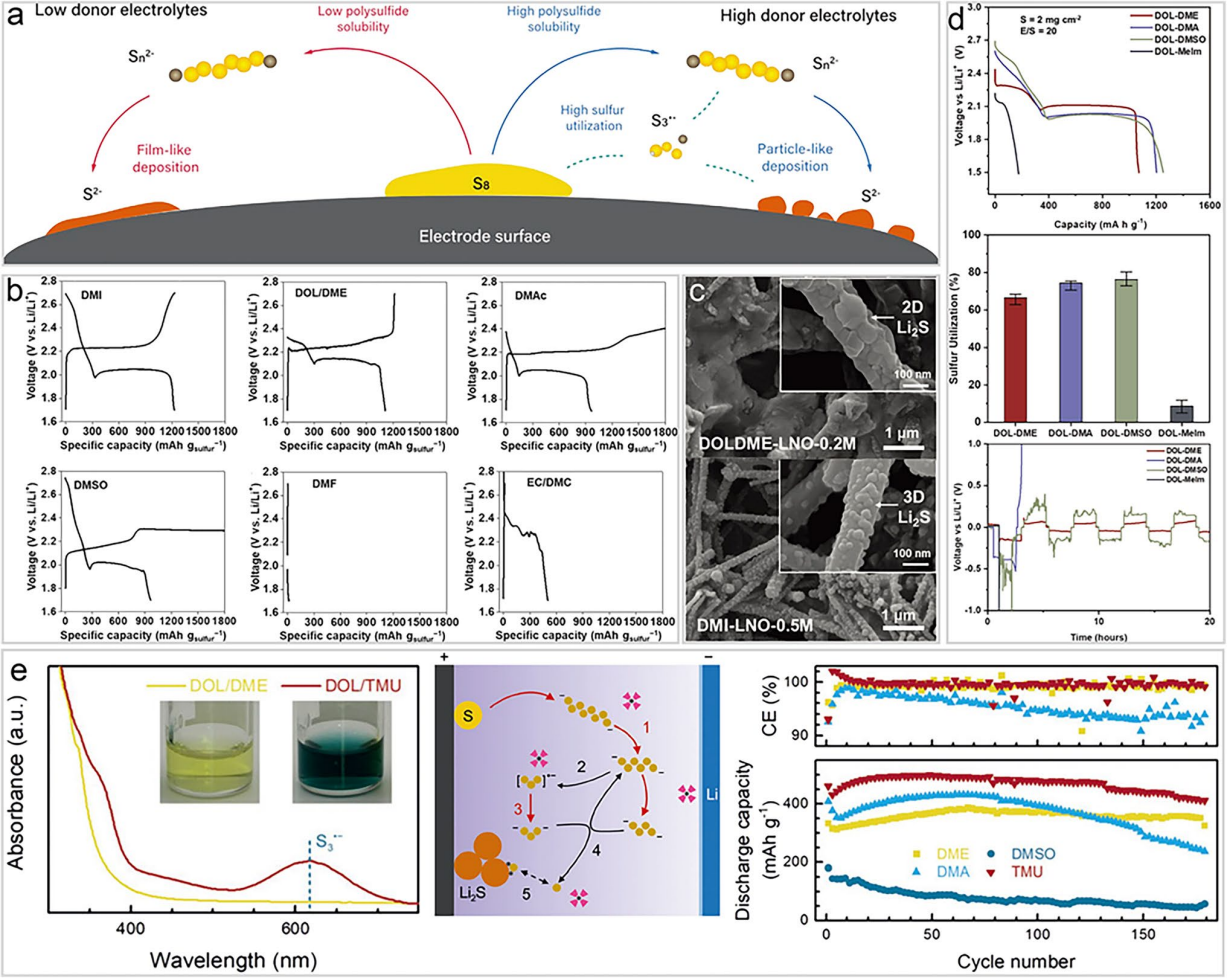
High-DN solvents and co-solvents to promote the generation of ${\text{S}}_{3}^{\bullet-}$/${\text{LiS}}_{3}^{\bullet}$ radicals. **a** Schematic illustration of the lithiation process of low and high donor electrolytes in LSBs [32]. Copyright 2020, John Wiley and Sons. **b** Charge–discharge profiles of LSBs with different electrolytes [100]. Copyright 2020, John Wiley and Sons. **c** SEM images of Li_2_S deposits on carbon electrodes [100]. Copyright 2020, John Wiley and Sons. **d** Discharge profiles and sulfur utilizations of sulfur cathode, and galvanostatic cycling of lithium symmetric cells in four blended electrolytes [42]. Copyright 2019, John Wiley and Sons. **e** The left: UV–Vis spectra of Li_2_S_6_ solution in different solvents, the middle: properties, mechanism and performance of TMU-based electrolyte, the right: cycling performance of Li/polysulfide in different solvent of coin cells [43]. Copyright 2018, John Wiley and Sons

High-DN solvents (such as DMA, DMSO, DMF) refer to the use of only a high-DN solvent (such as DMSO) as an electrolyte solvent, which show significant potential in LSBs due to their higher solubility for polysulfides than the traditional DOL/DME electrolytes, enabling high-energy-density LSBs under lean E/S ratio conditions. In addition, high-DN electrolytes facilitate polysulfide disproportionation and dissociation reactions, producing the intermediate ${\text{S}}_{3}^{\bullet-}$ radicals (Fig. 5a) [32]. These intermediates act as redox mediators, accelerating electrochemical reactions and improving sulfur utilization. Additionally, owning to the mediated role of ${\text{S}}_{3}^{\bullet-}$ radicals, high-DN electrolytes promote the deposition of Li_2_S in a 3D particle-like morphology, avoiding the passivation issues caused by insulating films, thereby maintaining electrode conductivity and extending cycle life.

Unfortunately, high-DN solvents have poor lithium metal compatibility and high viscosity, which lead to frequent side reactions and low ionic conductivity respectively, thereby limiting battery cyclability and rate ability. For example, those solvents containing strongly polar C=O or C–N bonds, such as DMA and DMF, readily react with the strongly reducing Li metal, leading to the formation of decomposition products [42]. Among them, 1,3-dimethyl-2-imidazolidinone (DMI), as a high-DN solvent, rarely exhibits excellent performance in LSBs (Fig. 5b) [100], thanks to its unique molecular structure and compatibility with the metallic Li anode. By replacing the oxygen atoms in ethylene carbonate (EC) with nitrogen, DMI stabilizes the carbonyl group, reducing its reactivity with polysulfides. It also promotes the formation and stabilization of ${\text{S}}_{3}^{\bullet-}$ radicals, activating additional reaction pathways that enhance sulfur utilization. Furthermore, DMI encourages the deposition of Li_2_S in a 3D particle form, preventing electrode passivation (Fig. 5c) [100]. However, it still needs to be used in conjunction with LiNO_3_ additives to further stabilize the metallic Li anode interface, reduce the shuttle effect, and improve cycling performance.

It should be pointed out here that in addition to being observed through SEM electron microscopy, Li_2_S 3D or 2D deposition can also be judged through the easier-to-achieve current curve fitting of the deposition process, according to Armstrong, Fleischmann and Thirsk (AFT) and Bewick, Fleischmann and Thirsk (BFT) models [108], or Avrami equation [43, 109, 110]. Avrami equation is described as: $Y(t) = 1 - \exp ( - \kappa t^{n} )$, where $Y(t)$ is the transformed volume fraction, $\kappa$ is rate constant, *n* is Avrami exponent, *t* is time. When *n* = 2, it corresponds to 2D instantaneous nucleation, that is, it becomes the BFT model, and when *n* = 3, it corresponds to 3D instantaneous nucleation, that is, it becomes the AFT model. The Avrami equation can be used to further explore the regulatory effect of trisulfur radicals on Li_2_S deposition.

To overcome the shortcomings of high-DN solvents, researchers have proposed a co-solvent strategy rationally. By the way, a high-DN solvent is mixed with a traditional ether-based solvent (such as DOL) in equivolume to combine the advantages of both solvents, thereby lowering its corrosiveness toward metallic Li while retaining its advantages in enhancing polysulfide solubility and promoting reactions. This solvent system significantly improves the cycling stability of the battery and enhances sulfur utilization, even under low E/S ratios. Furthermore, the co-solvent strategy optimizes the solvent combination to promote the formation of particulate Li_2_S deposits, preventing electrode passivation and further improving battery performance (Fig. 5d) [42].

In particular, the high-DN solvent tetramethylurea (TMU) that has good compatibility with the metallic Li anode significantly enhances the performance of LSBs when used in combination with the traditional ether-based solvent DOL (Fig. 5e) [43]. In the DOL/TMU electrolyte environment with 1.0 M LiTFSI and 0.30 M LiNO_3_ as the salts, ${\text{S}}_{3}^{\bullet-}$ radicals still exhibit visible blue color because of the high stability of high-DN solvent against ${\text{S}}_{3}^{\bullet-}$ radicals, indicating ${\text{S}}_{3}^{\bullet-}$ radicals exist in practical LSB system without being affected by lithium salts. TMU improves polysulfide solubility, promotes the generation of ${\text{S}}_{3}^{\bullet-}$ radicals, and activates multiple reaction pathways, thereby increasing the battery's efficiency and energy density. Additionally, ${\text{S}}_{3}^{\bullet-}$ radicals of TMU facilitate the 3D deposition of Li_2_S, preventing electrode passivation and enhancing specific capacity and cycling stability. Under the high E/S ratios, this co-solvent system significantly boosts battery performance, providing higher energy density and longer cycle life. However, extended cycling is limited by electrolyte depletion under harsh conditions, necessitating consideration of anode electrolyte consumption when reducing the E/S ratio in LSBs.

### High-DN Solvent Additives

**Fig. 6:**
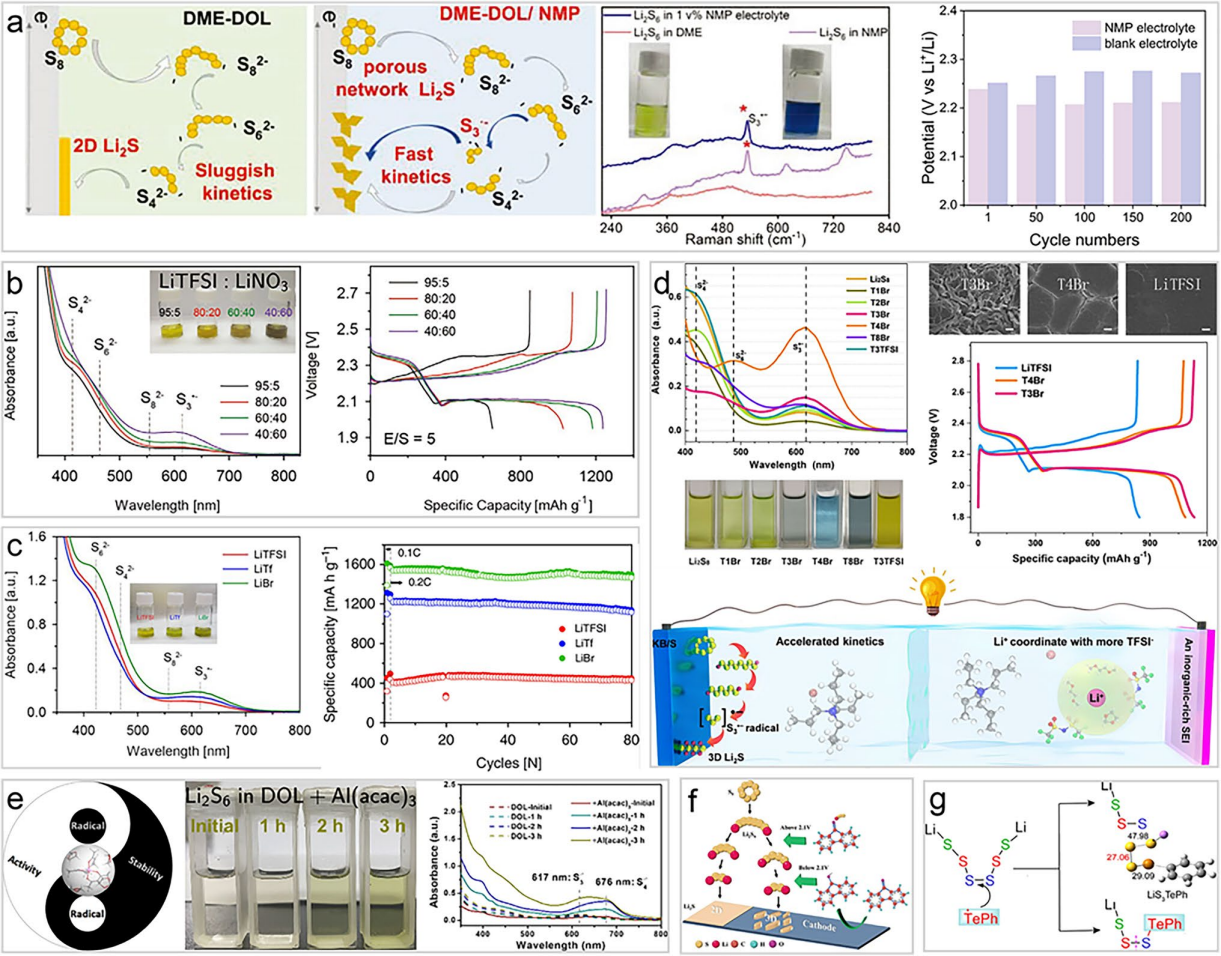
High-DN solvent and anion additives to promote generation of ${\text{S}}_{3}^{\bullet-}$/${\text{LiS}}_{3}^{\bullet}$ radicals. **a** The first and second: schematic of Li_2_S nucleation behaviors, the third: Raman of Li_2_S_6_ solutions, the fourth: Li_2_S oxidation overpotential of cells [101]. Copyright 2022, John Wiley and Sons. **b** The left: UV–Vis spectra of the LiPSs in the co-salt electrolytes, the right: discharge/charge profiles of LSB cells [111]. Copyright 2020, John Wiley and Sons. **c** UV–Vis spectra of Li_2_S_8_ solutions and capacities comparison of cells [102]. Copyright 2019, Springer Nature. **d** The upper left: UV–Vis spectra of the Li_2_S_8_ solution with the different salts, the upper right: SEM images of the cycled Li anodes, and corresponding discharge/charge profiles, the bottom: schematic of T3Br’s influence mechanism [56]. Copyright 2023, John Wiley and Sons. **e** The left: schematic of balancing stability and activity of ${\text{S}}_{3}^{\bullet-}$ radicals by metal complexes, the middle: time dependent photographs, and the right: UV–Vis spectra of Li_2_S_6_ with Al(acac)_3_ [55]. **f** Schematic of organic additives regulating LSB reactions [103]. Copyright 2023, Elsevier. **g** Simulation results of LiPSs reacting with organic additives [104]. Copyright 2022, John Wiley and Sons

High-DN solvent additive refers to that the volume of high-DN solvent in the electrolyte is relatively small (generally about 1% of the traditional ether-based solvent electrolyte). A small amount of high-DN solvent can effectively stabilize the ${\text{S}}_{3}^{\bullet-}$ radicals and avoid the corrosion of metallic Li anode at high concentration. In addition, because the proportion of high-DN solvents is very low, the side reaction caused by high concentration is avoided, and the metallic Li anode compatibility is significantly improved. Liang and co-workers proposed using high-DN solvent such as N-methyl-2-pyrrolidone (NMP) as electrolyte additives (< 1 vol%) [101]. This strategy minimizes the direct reactions between the solvent and the metallic Li anode, preventing corrosion of the metallic Li, while retaining the solvent’s advantage in promoting and stabilizing ${\text{S}}_{3}^{\bullet-}$ radicals (Fig. 6a). At low concentrations, NMP preferentially coordinate with Li^+^ ions, forming a stable solvated layer that inhibits corrosion of the metallic Li. Simultaneously, it enhances the 3D nucleation of Li_2_S, decreasing oxide reaction energy barrier and significantly improving sulfur conversion efficiency and the reversibility of the battery. This strategy not only reduces side reactions but also improves the cycling stability and capacity retention of LSBs, significantly extending the battery’s lifespan and providing an efficient and stable solution for the optimization of LSB electrolytes with a significant content of ${\text{S}}_{3}^{\bullet-}$ radicals. However, considering that high-DN solvents may affect the stability of the metallic Li anode, especially under lean electrolyte conditions, it is necessary to explore other methods to stabilize ${\text{S}}_{3}^{\bullet-}$ radicals.

### High-DN Anion Supporting Electrolytes or Electrolyte Additives

In addition to high-DN solvents, high-DN anion supporting electrolytes or electrolyte additives, that is, adding a small amount of salts (usually more than 0.5 M for supporting electrolytes and less than 0.1 M for electrolyte additives) with high-DN anions (such as ${\text{Br}}^{-}$) to traditional ether-based solvent electrolytes, can also effectively stabilize ${\text{S}}_{3}^{\bullet-}$ radicals through their strong coordination ability. This strategy provides a more stable and efficient solution without the need for additional protection of the metallic Li anode, offering a new direction for the optimization of LSBs. Compared with low-DN anions (such as the commonly used lithium salt TFSI^–^, DN = 5.4 kcal mol^–1^), high-DN anions, such as Br^−^ (DN = 33.7 kcal mol^–1^) and ${\text{NO}}_{3}^{-}$ (DN = 22.2 kcal mol^–1^), can enhance the solubility of LiPSs due to their strong electron-donating ability, suppress the passivation of the metallic Li anode, and promote the 3D particulate deposition of Li_2_S.

Chu *et al.* find that the concentration of the ${\text{S}}_{3}^{\bullet-}$ radicals is elevated with the higher content LiNO_3_ as one of the mixed lithium salts (Fig. 6b) [111], demonstrating the role of 0.6 M ${\text{NO}}_{3}^{-}$ with higher DN on stabilizing ${\text{S}}_{3}^{\bullet-}$ radicals. The results also indicate that the ${\text{S}}_{3}^{\bullet-}$ radicals still exhibit visible blue color in the traditional ether-based solvents containing high-DN anions. Based on its high electron-donating ability, the full cell could achieve a high sulfur utilization, and the capacity could reach above 1200 mA h g^−1^. Similarly, this research team also observed that the concentration of ${\text{S}}_{3}^{\bullet-}$ radicals is increased with increasing the high-DN ${\text{Br}}^{-}$ content in the traditional ether-based solvent electrolyte (Fig. 6c) [102]. These high-DN ${\text{Br}}^{-}$ stabilize the ${\text{S}}_{3}^{\bullet-}$ radicals, improving sulfur conversion efficiency and reaction kinetics, which enhances the reversibility and cycling stability of the battery. Furthermore, these high-DN salt anions promote the 3D deposition of Li_2_S, reduce electrode passivation, decrease polarization, and further enhance cycling performance and capacity retention.

Building on the important role of high-DN ${\text{Br}}^{-}$ anions in stabilizing the generation of ${\text{S}}_{3}^{\bullet-}$ radicals, Meng *et al.* further promoted the generation of ${\text{S}}_{3}^{\bullet-}$ radicals used 0.1 M quaternary ammonium salts (QASs) with tetra-alkyl ammonium cations (defined as T[*x* + 1]^+^, *x* denoting the number of –(CH_2_)– units on single chain, and *x* = 0, 1, 2, 3, or 7) as an electrolyte additive by manipulating the cations [56]. Figure 6d shows that QASs with symmetric carbon chains of specific lengths are more effective at triggering the generation of ${\text{S}}_{3}^{\bullet-}$ radicals, particularly the T3^+^ and T4^+^ structures. Among them, the T4Br additive provides the best stabilization of ${\text{S}}_{3}^{\bullet-}$, causing the polysulfide solution to exhibit a distinct blue color. Notably, T4^+^ paired with the low-DN TFSI^–^ anion to form T4TFSI cannot trigger the generation of ${\text{S}}_{3}^{\bullet-}$ radicals, indicating that the cation and high-DN anion have a synergistic coupling effect in promoting the generation of T4^+^. Furthermore, since the cation QAS promotes the formation of the solid electrolyte interface (SEI) on the metallic Li anode by altering the solvated structure of Li^+^, T3Br exhibits the best protection of the metallic Li anode, resulting in superior electrochemical performance in LSBs. This further underscores the importance of the synergistic effect between cations and high-DN anions in enhancing the performance of LSBs based on ${\text{S}}_{3}^{\bullet-}$ radicals’ catalysis/mediation and metallic Li anode protection. This strategy provides new insights for further optimizing electrolyte formulations for LSBs, particularly by combining large cations and high-DN anions to improve battery performance.

Although stabilizing ${\text{S}}_{3}^{\bullet-}$ radicals is crucial for improving the performance of LSBs, it needs to be done within a certain limit, *i.e.*, excessive stabilization can cause the radicals to lose their electrochemical activity, thus reducing the utilization of active materials in the battery. To balance the electrochemical activity and stability of ${\text{S}}_{3}^{\bullet-}$ radicals in LSBs, Zhao and co-workers proposed adding 0.04 M Al(acac)_3_ to a low DOL solvent, forming an Al(acac)_3_ complex through the ion–dipole interaction between DOL and Al^3+^ (Fig. 6e) [55]. In this solvated metal complexes, the oxygen atoms in DOL donate electrons to Al^3+^, weakening the attraction of Al^3+^ to sulfur radicals (*i.e.*, ${\text{S}}_{3}^{\bullet-}$ and ${\text{S}}_{4}^{{\bullet}-}$). This allows the radicals to maintain their reductive activity while avoiding over-stabilization that would lead to a loss of activity. This mechanism ensures the efficient conversion of sulfur radicals in LSBs and other multielectron transfer systems, while maintaining long-term stability and optimizing the performance and utilization of active materials. It is worth mentioning that this research proposed the ${\text{S}}_{4}^{{\bullet}-}$ stability mechanism for the first time, and its UV–Vis absorption peak at 676 nm and Raman shift at 517 cm^–1^ sets it apart from ${\text{S}}_{3}^{\bullet-}$ at 617 nm and 535 cm^–1^, respectively. The work has important reference significance for the development of other free radicals (*viz.* ${\text{S}}_{2}^{{\bullet}-}$, ${\text{S}}_{4}^{{\bullet}-}$, ${\text{S}}_{5}^{{\bullet}-}$, ${\text{S}}_{6}^{{\bullet}-}$, ${\text{S}}_{7}^{{\bullet}-}$, and ${\text{S}}_{8}^{{\bullet}-}$) for their application in LSBs.

In addition to inorganic electrolyte additives, Zhao's team also used 0.1 M fluorenone (FL) as an organic electrolyte additive to stabilize ${\text{S}}_{3}^{\bullet-}$ radicals [103]. As shown in Fig. 6f, carbonyl groups of FL can capture and stabilize ${\text{S}}_{3}^{\bullet-}$ radicals through its electron-accepting ability, which promotes the three-dimensional deposition of Li_2_S, reducing the internal resistance of the battery and improving capacity and cycle stability. In addition, FL can be reduced to ${\text{FL}}^{{\bullet}-}$ radical within the operating voltage of LSBs. As an electron transfer medium, it can accelerate the reduction process of Li_2_S_4_ → Li_2_S and the oxidation activation energy of Li_2_S, thus improving the kinetic reaction and battery performance.

Similarly, Zhang *et al.* used 0.05 M diphenyl ditelluride (DPDTe) as an organic electrolyte additive to promote the generation of ${\text{S}}_{3}^{\bullet-}$ radicals in LSBs [104]. During the discharge process, DPDTe is electrochemically reduced to form the PhTe^•^ free radical, which can undergo rapid radical exchange with Li_2_S_6_, generating more electrochemically reactive ${\text{LiS}}_{3}^{\bullet}$ and LiS_3_TePh (Fig. 6g). The former has been extensively discussed for its catalytic mediation role in LSBs, while the latter is further reduced to form LiSTePh and Li_2_S_2_. The Li_2_S_2_ is continuously attacked by ${\text{PhTe}}^{\bullet}$ radicals, generating LiSTePh as an intermediate and ultimately Li_2_S as the final product. The formed LiSTePh can be easily lithiated to form Li_2_S and regenerate ${\text{PhTe}}^{\bullet}$ radicals, completing a Te-radical-mediated catalytic cycle. This dual-free-radical synergistic effect based on organic telluride electrolyte additives enables LSBs to exhibit impressive cycling stability and rate performance.

### Metal Compound Catalysts

**Fig. 7:**
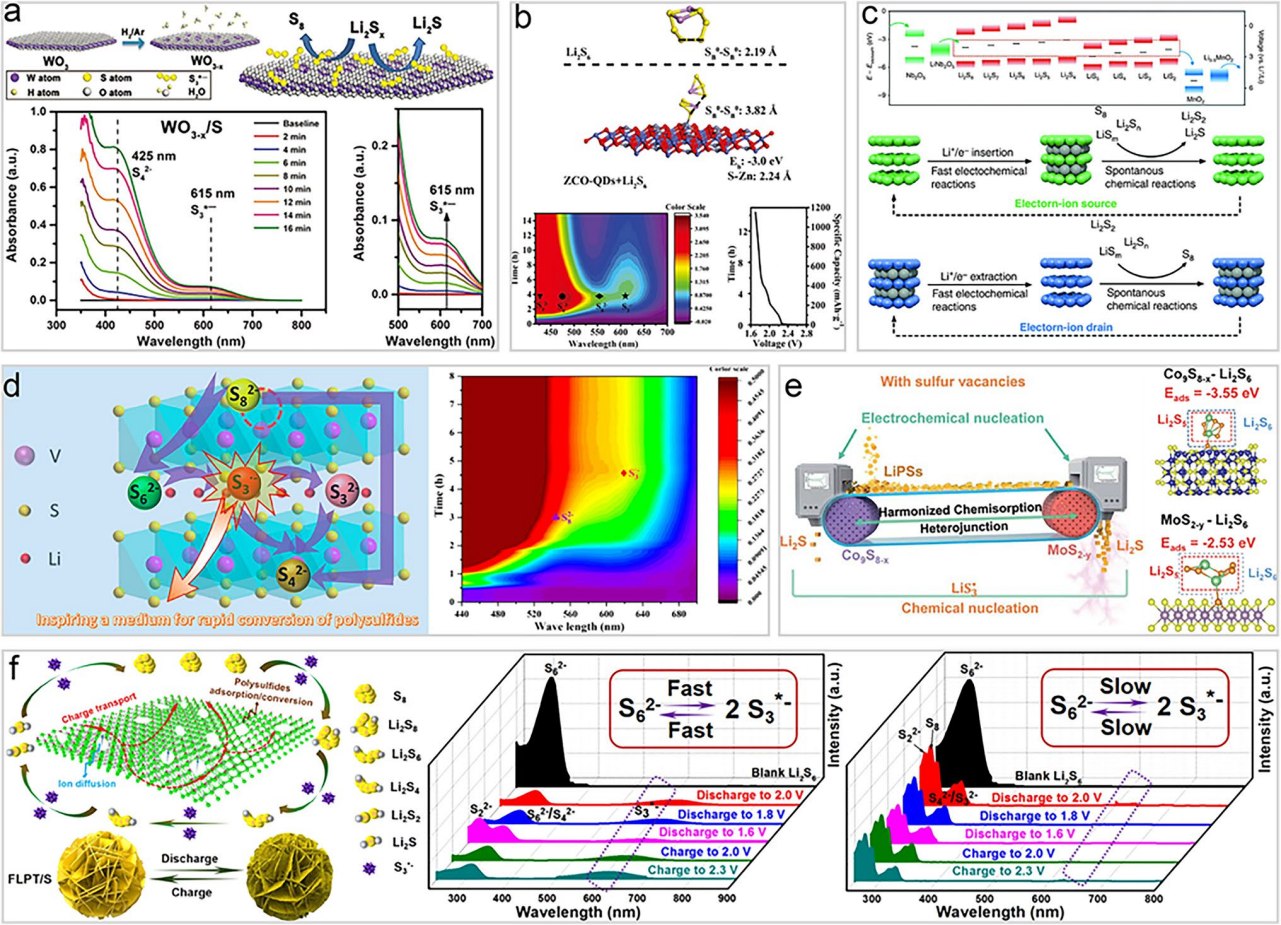
Metal compound catalysts to promote the generation of ${\text{S}}_{3}^{\bullet-}$/${\text{LiS}}_{3}^{\bullet}$ radicals. **a** The top: schematic of WO_3−*x*_ and the conversion of Li_2_S_x_ on its surface, the bottom: time dependent UV–Vis spectra of sulfur cathodes with WO_3−*x*_ [53]. Copyright 2018, John Wiley and Sons. **b** The top: optimized geometric configurations of Li_2_S_6_ adsorbed on ZCO-QDs, the bottom: contour maps of *in situ* UV–Vis spectra, and the corresponding discharge profiles of ZCO-QDs based cathode [113]. Copyright 2021, John Wiley and Sons. **c** The top: electron transfer with electron–ion reservoirs. the bottom: schematic of reactions coupling to enhance kinetics during operation [81]. Copyright 2020, Springer Nature. **d** The left: schematic of the adsorption-catalytic behavior of polysulfides on VS_2−*x*_, the right: *in situ* UV–Vis spectra of the VS_2−*x*_ based cathodes [105]. Copyright 2022, American Chemical Society. **e** The left: catalytic mechanism of sulfur vacancy heterojunctions, the right: calculated adsorption energies of Li_2_S_6_ on MoS_2−*y*_ and Co_9_S_8−*x*_ crystals [106]. Copyright 2022, Royal Society of Chemistry. **f** The left: schematic of the interaction of LiPSs on MXenes, the right: UV–Vis spectra of cathodes at different states [107]. Copyright 2019, American Chemical Society

Metal oxide catalysis of sulfur chemical conversion reactions plays an important role in LSBs, particularly oxygen-deficient metal oxides, which exhibits excellent catalytic performance in accelerating polysulfide conversion reactions [112]. Lin et al*.* prepared oxygen-deficient tungsten oxide (WO_3−*x*_) and used it as a sulfur host for LSBs. Oxygen defects reduce the oxidation state of metal oxides and promote the formation and stabilization of highly active ${\text{S}}_{3}^{\bullet-}$ radicals on their surfaces, which was verified by UV–Vis (Fig. 7a) [53]. Moreover, oxygen deficiency creates more active sites, enhancing the metal oxide surface's ability to adsorb polysulfides, thereby suppressing the shuttle effect. As a result, WO_3−*x*_ improved the kinetics of polysulfide conversion and significantly enhanced the cycle stability and high-rate performance of LSBs.

Oxygen-deficient metal oxides, in addition to their traditional roles in polysulfide adsorption and catalysis, may also stabilize ${\text{S}}_{3}^{\bullet-}$ radicals. However, the underlying mechanism still requires further exploration. Liu *et al.* found that the ZnCo_2_O_4_ composite oxide can significantly promote the generation of ${\text{LiS}}_{3}^{\bullet}$ radicals (Fig. 7b) [113]. DFT calculations show that the abundant metal active sites on the surface of ZnCo_2_O_4_ adsorb Li_2_S_6_ through metal–S bonds, reducing its stability and promoting its cleavage into ${\text{LiS}}_{3}^{\bullet}$ radicals. These radicals can further consume solid sulfur through reactions such as 2 ${\text{S}}_{3}^{\bullet-}$ + 1/4S_8_ → ${\text{S}}_{8}^{2-}$, generating other reducible polysulfides, thereby improving sulfur utilization and the cycle stability of the battery. Notably, although oxygen vacancies were also detected on the surface of ZnCo_2_O_4_, they were not considered to stabilize the ${\text{S}}_{3}^{\bullet-}$ radicals. Instead, they enhance the adsorption of Li_2_S_4_, thereby promoting the efficiency of its reduction reaction.

In addition to adsorption and catalytic effects, metal oxides can also catalyze sulfur chemical transformations through their electron–ion source and drain functions (Fig. 7c) [81]. Based on molecular orbital theory, Lu *et* *al.* suggested that the band gap center (BGC, derived from the midpoint of HOMO and LUMO energy levels) of the tetra-coordinated lithium polysulfides (Li_2_S_n_-4DOL) is higher than that of the bi-coordinated lithium sulfur radicals (${\text{LiS}}_{\text{n}}^{\bullet}$-2DOL), indicating that the reduction of ${\text{LiS}}_{\text{n}}^{\bullet}$-2DOL occurs prior to Li_2_S_n_-4DOL. Once ${\text{LiS}}_{\text{n}}^{\bullet}$-2DOL is consumed, Li_2_S_n_-4DOL spontaneously converts to regenerate ${\text{LiS}}_{\text{n}}^{\bullet}$-2DOL. Integrating this understanding with band theory, they employed a mixed metal oxide catalyst (Nb_2_O_5_/MnO_2_) featuring both high and low BGC values (corresponding to the valence band and conduction band centers) as a cathode catalyst for LSBs. During the discharge process, lithiated LiNb_2_O_5_ acts as an electron and ion source, providing electrons and Li^+^ ions to ${\text{LiS}}_{\text{n}}^{\bullet}$-2DOL, thereby accelerating the electrochemical reduction of Li_2_S_n_/${\text{LiS}}_{\text{n}}^{\bullet}$ to Li_2_S. Conversely, during the charging process, delithiated MnO_2_ acts as an electron and ion drain, extracting electrons and Li^+^ ions from Li_2_S_n_/${\text{LiS}}_{\text{n}}^{\bullet}$, thus promoting the oxidation of Li_2_S_n_/${\text{LiS}}_{\text{n}}^{\bullet}$ to S_8_. Notably, lithium sulfur radicals (including ${\text{LiS}}_{3}^{\bullet}$ radicals), which arise from the homolytic or heterolytic cleavage of Li_2_S_x_, serve as critical intermediates in this catalytic process. Although their concentration may be limited due to chemical equilibrium, the electron–ion source and drain catalyst significantly enhances their role in facilitating these reactions.

Metal sulfide catalysts are also widely applied in LSBs to promote the electrochemical conversion and reaction kinetics of sulfur species [114]. Wang *.* applied sulfur-defect-enriched VS_2_ nanosheets (VS_2−*x*_) as catalysts in LSBs and found that both VS_2−*x*_ and its lithiation intermediate Li_y_VS_2−*x*_ significantly enhance the content of ${\text{S}}_{3}^{\bullet-}$ radicals by promoting the dissociation of ${\text{S}}_{6}^{2-}$(Fig. 7d) [105]. These free radicals facilitate the ring-opening reaction of cyclo-S_8_, accelerating the conversion of sulfur, reducing the shuttle effect of polysulfides, and improving the sulfur utilization, cycling stability, and reaction kinetics of the battery.

Notably, the introduction of sulfur vacancies also ensures the long-term stability of the catalyst, allowing it to continuously exert its catalytic effect during the charge and discharge processes. Wei and co-workers also introduced sulfur vacancy catalysts (Co_9_S_8_/MoS_2_ heterojunction) in LSBs to promote the content of ${\text{S}}_{3}^{\bullet-}$ radicals (Fig. 7e) [106]. In contrast, they proposed that the ${\text{LiS}}_{3}^{\bullet}$ radicals are not directly dissociated from Li_2_S_6_ adsorbed on the sulfide, but rather, when Li_2_S_6_ interacts with sulfur vacancies, one of the sulfur atoms in the Li_2_S_6_ molecule is asymmetrically adsorbed to the sulfur vacancy, leading to the formation of the Li_2_S_5_ intermediate, according to theoretical calculations. Li_2_S_5_ is a relatively unstable and highly reactive species, which can further convert into ${\text{LiS}}_{3}^{\bullet}$ radicals and other polysulfide species. This study provides new insights into the use of vacancy defects in metal compounds to stabilize ${\text{S}}_{3}^{\bullet-}$ radicals in LSBs.

Special metallic compound MXene, as a class of emerging two-dimensional transition metal carbides or nitrides [115], have gradually become important materials in LSBs due to their unique nanostructures, excellent conductivity, and surface chemical properties [116, 117]. MXene materials such as Ti_3_C_2_T_*x*_ have abundant polar surface sites that can effectively adsorb and catalyze the transformation of polysulfides, thereby improving the performance of LSBs [118–120]. Xiao *et al.* prepared a flower-like porous MXene material Ti_3_C_2_T_*x*_ (FLPT) and applied it to the cathode of LSBs (Fig. 7f) [107]. The surface of FLPT is rich in polar groups, such as hydroxyl and oxygen, which, through Lewis acid–base interactions, strongly adsorb polysulfides (especially ${\text{S}}_{6}^{2-}$). According to the enhanced characteristic adsorption at 617 nm through in situ UV–Vis spectroscopy, this strong adsorption can effectively promote the generation and stabilization of ${\text{S}}_{3}^{\bullet-}$ radicals, thereby accelerating the sulfur species transformation in LSBs. Furthermore, the high conductivity and nanosheet structure of FLPT further promote the rapid charge transfer and efficient ion transport, allowing the ${\text{S}}_{3}^{\bullet-}$ radicals to quickly participate in electrochemical reactions on the electrode surface. The three-phase interface effect (FLPT support, sulfur species, and electrolyte) also enhances the enrichment and stabilization of ${\text{S}}_{3}^{\bullet-}$ radicals, further facilitating the oxidation–reduction reactions of sulfur. Therefore, FLPT materials not only enhance the stability of ${\text{S}}_{3}^{\bullet-}$ radicals through strong chemical adsorption and rapid charge transfer mechanisms but also optimize the electrochemical performance of LSBs, improving the battery's capacity and cycle stability. This mechanism of stabilizing ${\text{S}}_{3}^{\bullet-}$ radicals provides new insights into the development and application of MXene materials in LSBs, highlighting their potential in advancing the performance and practical deployment of next-generation energy storage systems.

### Carbon-Based Catalysts

Carbon-based catalysts, with their excellent conductivity, abundant active sites, and tunable surface chemistry, exhibit significant potential in LSBs [121, 122]. Generally, the higher specific surface area and more porous structure of carbon-based catalysts provide more surface and active sites for adsorption and better diffusion of LiPSs [121], including trisulfur radicals. Specially, carbon-based catalysts with heteroatom doping are crucial for anchoring trisulfur radicals and enhancing their stability. Note that the type and quantity of doped heteroatoms have different effects on the degree of graphitization [121], which has a positive correlation with electrical conductivity. For example, nitrogen doping can promote carbon graphitization, while oxygen doping generally does not favor graphitization. Therefore, the balance between electron conductivity and trisulfur radical stability should be considered for heteroatom doping of carbon-based catalysts.

**Fig. 8:**
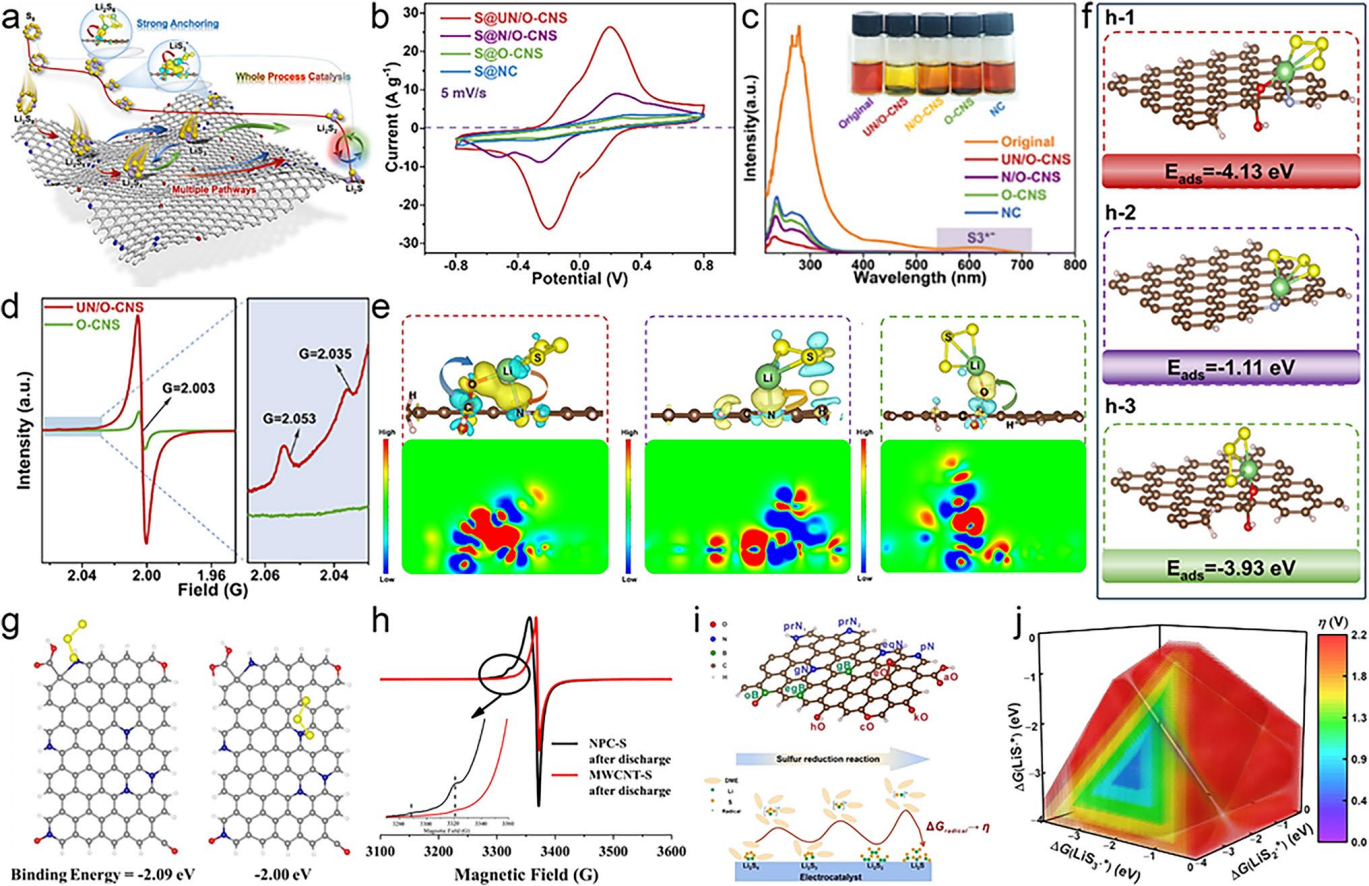
Carbon-based catalysts to promote the generation of ${\text{S}}_{3}^{\bullet-}$/${\text{LiS}}_{3}^{\bullet}$ radicals. **a** Schematic of strategies with carbon-based electrocatalyst [54], Copyright 2024, Elsevier. **b** CV curves of symmetric cells with Li_2_S_6_ electrolyte [54]. Copyright 2024, Elsevier. **c** UV–Vis spectra of materials after adsorption of Li_2_S_6_. Copyright 2024, Elsevier. **d** EPR spectra of carbon-based catalysts after discharge. Copyright 2024, Elsevier. **e** Electron density differences and **f** binding configurations of ${\text{LiS}}_{3}^{\bullet}$ on the different heteroatom-doped graphene [54]. Copyright 2024, Elsevier. **g** Optimized configurations of ${\text{S}}_{3}^{\bullet-}$ radicals absorbed on the N-doped carbon [123]. Copyright 2015, Elsevier. **h** ESR spectra of carbon/sulfur composite in the discharged state [123]. Copyright 2015, Elsevier. **i** Schematic of the reaction mechanism on the second discharge platform of LSBs with heteroatom-doped graphene [124]. Copyright 2022, John Wiley and Sons. **j** The overpotential with the adsorption Gibbs free energies of radicals [124]. Copyright 2022, John Wiley and Sons

Zhang and co-workers developed a carbon-based catalyst (UN/O-CNS) through heteroatom doping (such as N/O co-doping) [54], which not only effectively anchors ${\text{LiS}}_{3}^{\bullet}$ radicals (Fig. 8a), but also reduces the energy barriers of SRR and sulfur oxidation reactions (SOR) (Fig. 8b), thereby significantly enhancing the electrochemical conversion process. UV–Vis testing of the supernatant after UN/O-CNS adsorbed Li_2_S_6_ solution revealed that the peak intensities of polysulfide ions and ${\text{LiS}}_{3}^{\bullet}$ radicals were significantly reduced compared to the control group (Fig. 8c), indicating that the N/O dual active sites firmly anchor ${\text{LiS}}_{3}^{\bullet}$ radicals, preventing their aggregation or side reactions due to high reactivity. Post-cycling UN/O-CNS cathode materials displayed additional peaks at *G* = 2.035 and *G* = 2.053 (Fig. 8d) [54], further confirming the presence of anchored ${\text{LiS}}_{3}^{\bullet}$ radicals. Compared to models with pure N or O doping, the N/O co-doped model showed significant charge redistribution (Fig. 8e), with a substantial electron concentration at the triangular bond positions, implying stronger charge transfer and higher binding energy (Fig. 8f). Consequently, under the influence of its high specific surface area, porous structure, and synergistic catalytic active sites, UN/O-CNS achieves efficient capture of ${\text{LiS}}_{3}^{\bullet}$ radicals, stabilizes the radicals through triangular bonding, effectively suppresses the polysulfide shuttle effect, and enhances the conversion efficiency of sulfur species.

Chen *et al.* discovered that N-doped porous carbon (NPC) can effectively stabilize ${\text{S}}_{3}^{\bullet-}$ radicals [123]. N-doped carbon-based materials provide active sites such as pyrrolic-N and pyridinic-N. DFT calculations show that ${\text{S}}_{3}^{\bullet-}$ radicals preferentially interact with these N atoms, exhibiting low adsorption energies of −2.09 and −2.00 eV, respectively (Fig. 8g). ESR tests reveal that in the discharged NPC-S composites, in addition to carbon radicals (*g* = 2.0023), distinct signals of ${\text{S}}_{3}^{\bullet-}$ radicals (*g*_*y*_ = 2.0355, *g*_*z*_ = 2.0526) are present (Fig. 8h), further confirming the ability of NPC to capture ${\text{S}}_{3}^{\bullet-}$ radicals. This adsorption and stabilization mechanism of ${\text{S}}_{3}^{\bullet-}$ radicals by NPC significantly improves the cycling stability and Coulombic efficiency of LSBs, effectively suppressing the shuttle effect.

To elucidate the mechanism of heteroatom in carbon-based electrocatalyst, Feng *et al.* developed a heteroatom-doped carbon-based electrocatalytic model, using DFT calculations to analyze the impact of heteroatom doping on active sites on the carbon material surface [124]. The study showed that doping heteroatoms can effectively modulate the adsorption and conversion of lithium sulfur radicals ${\text{LiS}}_{\text{y}}^{\bullet}$ (*y* = 1–3) and short-chain Li_2_S_*x*_ (*x* = 1–4), significantly improving reaction efficiency (Fig. 8i). Furthermore, by using the adsorption energy of ${\text{LiS}}_{\text{y}}^{\bullet}$ radicals on the catalyst as a key descriptor, the study predicts the reaction pathway, rate-determining step, and overpotential (Fig. 8j). This research provides valuable theoretical insights into the mechanism of heteroatom-doped carbon-based electrocatalysts in promoting the generation of sulfur radicals including trisulfur radicals, contributing to the further optimization of LSBs performance. In recent years, machine learning has begun to be applied in the design of LSB materials. Feeding large amounts of computational data (such as reaction path, transition state, electronic structure changes of the active site) generated by DFT into machine learning models enables rapid prediction and screening of materials with excellent electrocatalytic properties [125]. This multidisciplinary approach can significantly improve the efficiency of developing materials that promote trisulfur radical generation and reduce the reliance on expensive experimental and computational resources.

Different from the adsorption mechanism of ${\text{S}}_{3}^{\bullet-}$ radicals, Kumar *et al.* proposed a grafting mechanism. Specifically, activated carbon cloth (ACC) with abundant carbon radicals serve as an effective sulfur host [126]. The dangling bond carbon radicals on ACC can couple with ${\text{S}}_{3}^{\bullet-}$ radicals, modulating the chemical conversion pathways and reaction kinetics of sulfur cathode. This radical grafting mechanism ultimately enhances the rate performance and cycling stability of sodium–sulfur batteries.

## Comprehensive Comparison of ${\mathbf{S}}_{\mathbf{3}}^{{\bullet}\mathbf{-}}$/${\mathbf{L}\mathbf{i}\mathbf{S}}_{\mathbf{3}}^{\bullet}$ Radicals Detection and Increment

### Advancing Detection Techniques

**Fig. 9:**
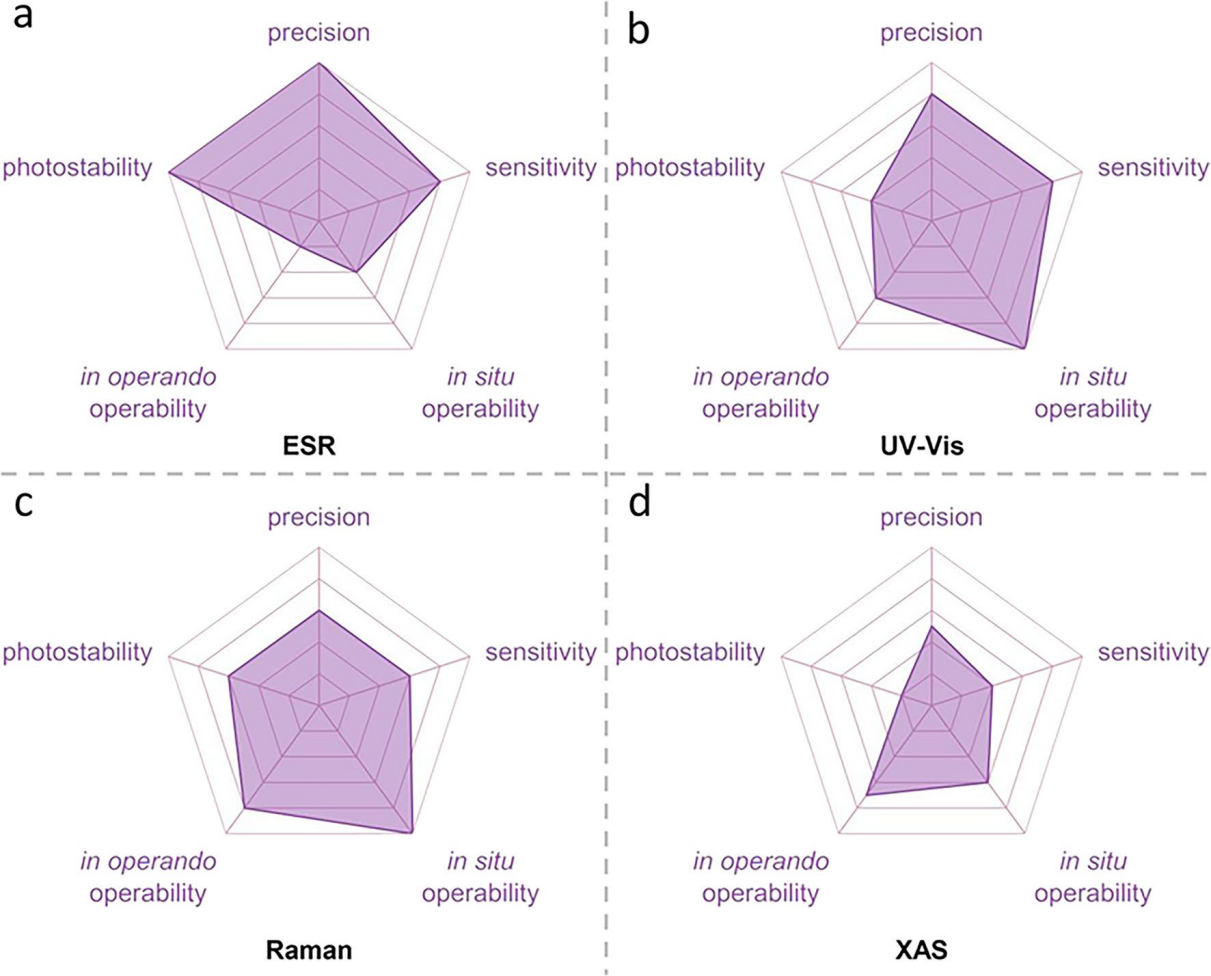
Radar map of optical testing technique with aspect of precision, photostability, *in operando* and *in situ* operability for **a** ESR, **b** UV–Vis, **c** Raman, and **d** XAS

In the detection of ${\text{S}}_{3}^{\bullet-}$/${\text{LiS}}_{3}^{\bullet}$ radicals in LSBs, the selection of spectroscopic techniques needs to consider multiple key factors, including precision, sensitivity, *in situ* operability, *in operando* operability, and photostability, as illustrated in radar map of Fig. 9. Photostability refers to the stability of the process in which polysulfides dissociate to form ${\text{S}}_{3}^{\bullet-}$/${\text{LiS}}_{3}^{\bullet}$ radicals under light sources, where higher stability indicates less interference from the light source on the radical signal. *In situ* operability involves real-time observation of the generation and behavior of ${\text{S}}_{3}^{\bullet-}$/${\text{LiS}}_{3}^{\bullet}$ radicals under operating conditions, while *in operando* operability further emphasizes the dynamic correlation between ${\text{S}}_{3}^{\bullet-}$/${\text{LiS}}_{3}^{\bullet}$ radicals behavior and the electrochemical performance of the battery.

ESR stands out due to its high precision and sensitivity, allowing direct detection of low-concentration ${\text{S}}_{3}^{\bullet-}$/${\text{LiS}}_{3}^{\bullet}$ radicals (Fig. 9a). It also exhibits good photostability, making it well-suited for detailed studies of the generation and transformation mechanisms of radicals. However, its demanding experimental conditions, complex equipment, and high costs limit its widespread application. In contrast, UV–Vis and Raman spectroscopy offer significant advantages in in situ and real-time *in operando* monitoring (Fig. 9b, c), with convenient operation that makes them suitable for studying the dynamic generation and transformation of ${\text{S}}_{3}^{\bullet-}$/${\text{LiS}}_{3}^{\bullet}$ radicals. However, these techniques have relatively lower sensitivity and light stability, with light sources potentially interfering with the radicals’ signal, making them more suitable as complementary methods. XAS is well-suited for exploring the electronic structure and chemical environment of ${\text{S}}_{3}^{\bullet-}$/${\text{LiS}}_{3}^{\bullet}$ radicals, with its high photon energy providing atomic-level resolution. However, its precision and sensitivity are moderate, and the high photon energy can lead to the decomposition of radicals or polysulfides, resulting in poor photostability and high equipment costs (Fig. 9d).

With the development of advanced spectroscopic techniques, many emerging methods have shown potential for ${\text{S}}_{3}^{\bullet-}$/${\text{LiS}}_{3}^{\bullet}$ radical research. For instance, time-resolved spectroscopy can dynamically capture the temporal processes of radical generation and transformation, providing key insights into radical reaction kinetics. Furthermore, two-photon spectroscopy and ultrafast laser spectroscopy offer high-resolution and short timescale observation, enabling the capture of short-lived radical states and providing new tools for elucidating their transient behaviors. Synchrotron X-ray spectroscopy further enhances the resolution and sensitivity of XAS, and when combined with in situ electrochemical cells, it allows for an in-depth correlation between ${\text{S}}_{3}^{\bullet-}$/${\text{LiS}}_{3}^{\bullet}$ radicals behaviors and electrochemical processes. Integrating multiple spectroscopic techniques, such as the combination of Raman with ESR or XAS with UV–Vis, can offer a more comprehensive analysis of the generation mechanisms and stabilization processes of ${\text{S}}_{3}^{\bullet-}$/${\text{LiS}}_{3}^{\bullet}$ radicals.

Each spectroscopic technique has unique characteristics in ${\text{S}}_{3}^{\bullet-}$/${\text{LiS}}_{3}^{\bullet}$ radical detection, and their selection requires a trade-off between precision, sensitivity, photostability, and operability depending on experimental needs. Moreover, with the introduction of advanced spectroscopic techniques and the realization of multitechnology synergies, future research is expected to systematically unravel the generation and stabilization mechanisms of ${\text{S}}_{3}^{\bullet-}$/${\text{LiS}}_{3}^{\bullet}$ radicals and their relationships with LSBs performance, providing crucial insights for LSB design and optimization.

### Constructing Catalytic System with High Content of ${\mathbf{S}}_{\mathbf{3}}^{{\bullet}\mathbf{-}}$/${\mathbf{L}\mathbf{i}\mathbf{S}}_{\mathbf{3}}^{\bullet}$ Radicals

**Fig. 10:**
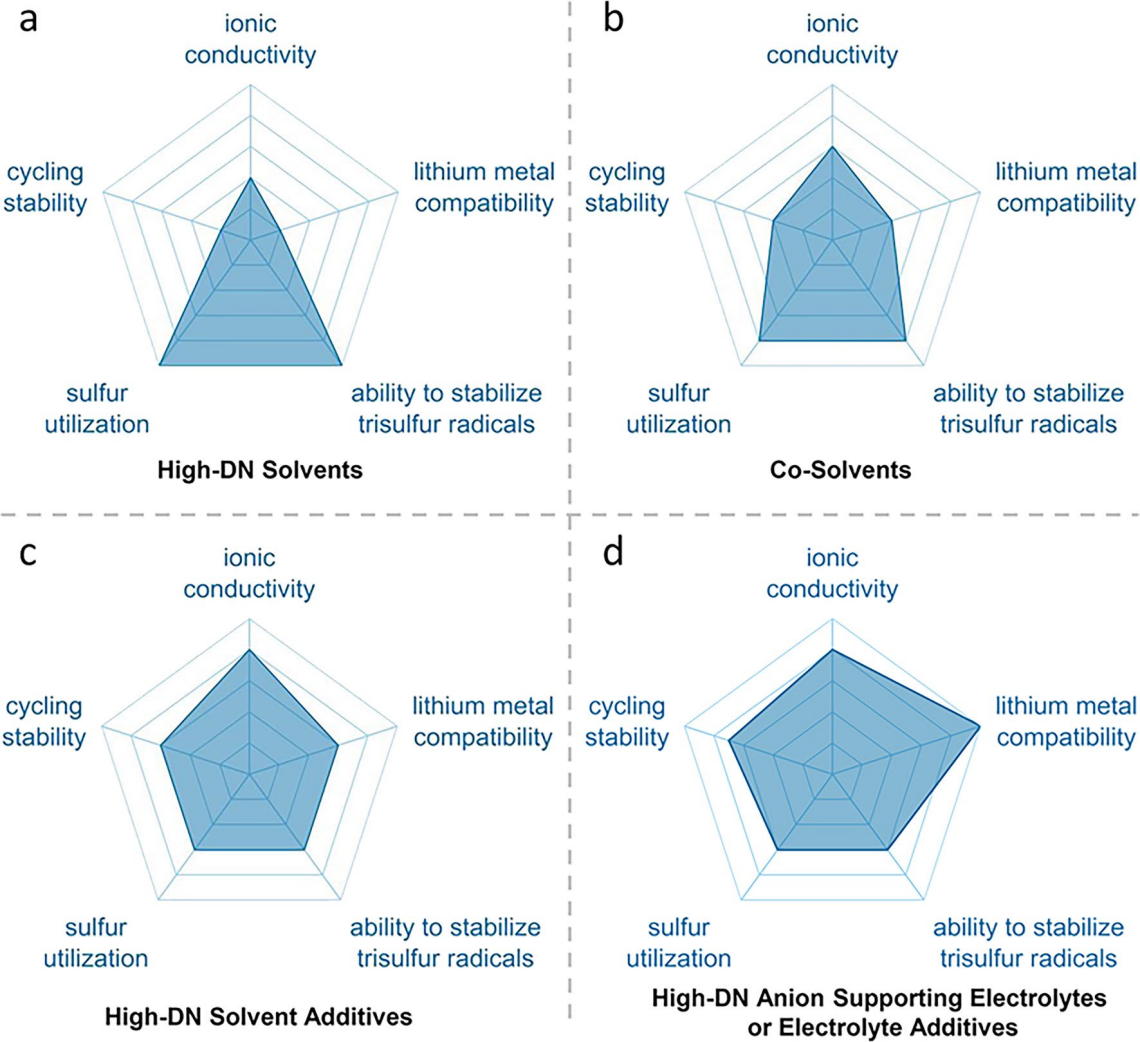
Radar map of homogeneous electrocatalyst strategy with aspect of ionic conductivity, cycling stability, sulfur utilization, ability to stabilize ${\text{S}}_{3}^{\bullet-}$/${\text{LiS}}_{3}^{\bullet}$ radicals and lithium metal compatibility for **a** high-DN solvents, **b** co-solvents, **c** high-DN solvent additives, and **d** high-DN anion supporting electrolytes or electrolyte additives

Different electrolyte strategies exhibit unique advantages and limitations in stabilizing ${\text{S}}_{3}^{\bullet-}$/${\text{LiS}}_{3}^{\bullet}$ radicals and improving the performance of LSBs (Fig. 10). High-DN solvents, with their strong coordination capabilities, significantly promote the dissociation of polysulfides and stabilize ${\text{S}}_{3}^{\bullet-}$/${\text{LiS}}_{3}^{\bullet}$ radicals (Fig. 10a), thereby enhancing sulfur utilization. However, their strong reactivity with metallic Li leads to poor compatibility with metallic Li anodes and reduced cycling stability. These issues can be mitigated through rational solvent selection, such as combining high-DN solvents with low-DN solvents to reduce corrosive interactions. Additionally, functional additives (such as LiNO_3_) can strengthen the SEI and enhance metallic Li protection.

Co-solvents, a mixture of high-DN solvents and traditional ether-based solvents, demonstrate a balanced performance, with good ionic conductivity, improved lithium compatibility, and enhanced cycling stability (Fig. 10b). However, the dilution of high-DN solvents reduces their ability to stabilize ${\text{S}}_{3}^{\bullet-}$/${\text{LiS}}_{3}^{\bullet}$ radicals. To optimize this strategy, adjusting the solvent ratio and viscosity can enhance polysulfide solubility and diffusion efficiency while further suppressing the shuttle effect. Furthermore, selecting low-viscosity ether solvents can improve ion transport, thereby boosting overall battery performance.

High-DN solvent additives, involving the addition of a small amount of high-DN solvents, effectively stabilize ${\text{S}}_{3}^{\bullet-}$/${\text{LiS}}_{3}^{\bullet}$ radicals while avoiding severe lithium corrosion associated with higher concentrations (Fig. 10c). This approach offers an excellent balance between stability and efficiency, making it suitable for applications requiring well-rounded performance. Additionally, combining this strategy with other functional additives, such as high-DN anions or lithium salts, can further enhance the comprehensive performance of the electrolyte.

High-DN anions supporting electrolyte or electrolyte additives, achieved by introducing salts with high-DN anions (such as ${\text{Br}}^{-}$) in traditional ether-based electrolytes, provide excellent metallic Li protection, significantly improving cycling stability and sulfur utilization (Fig. 10d). However, their direct stabilization effect on ${\text{S}}_{3}^{\bullet-}$/${\text{LiS}}_{3}^{\bullet}$ radicals is relatively limited. To address this, optimizing the solvent-salt ratio can improve ionic conductivity and polysulfide conversion efficiency. Furthermore, leveraging machine learning (ML) and computational simulations can accelerate the development of more effective additive combinations tailored for specific applications.

Therefore, integrating electrolyte engineering design into the discussion of electrolyte strategies offers a more systematic framework for optimizing LSBs performance. By combining solvent selection, additive design, viscosity control, and advanced computational techniques, it is possible to balance trisulfur radical stabilization, sulfur conversion efficiency, and cycling stability while advancing the technological breakthrough and practical implementation of LSBs.

**Fig. 11:**
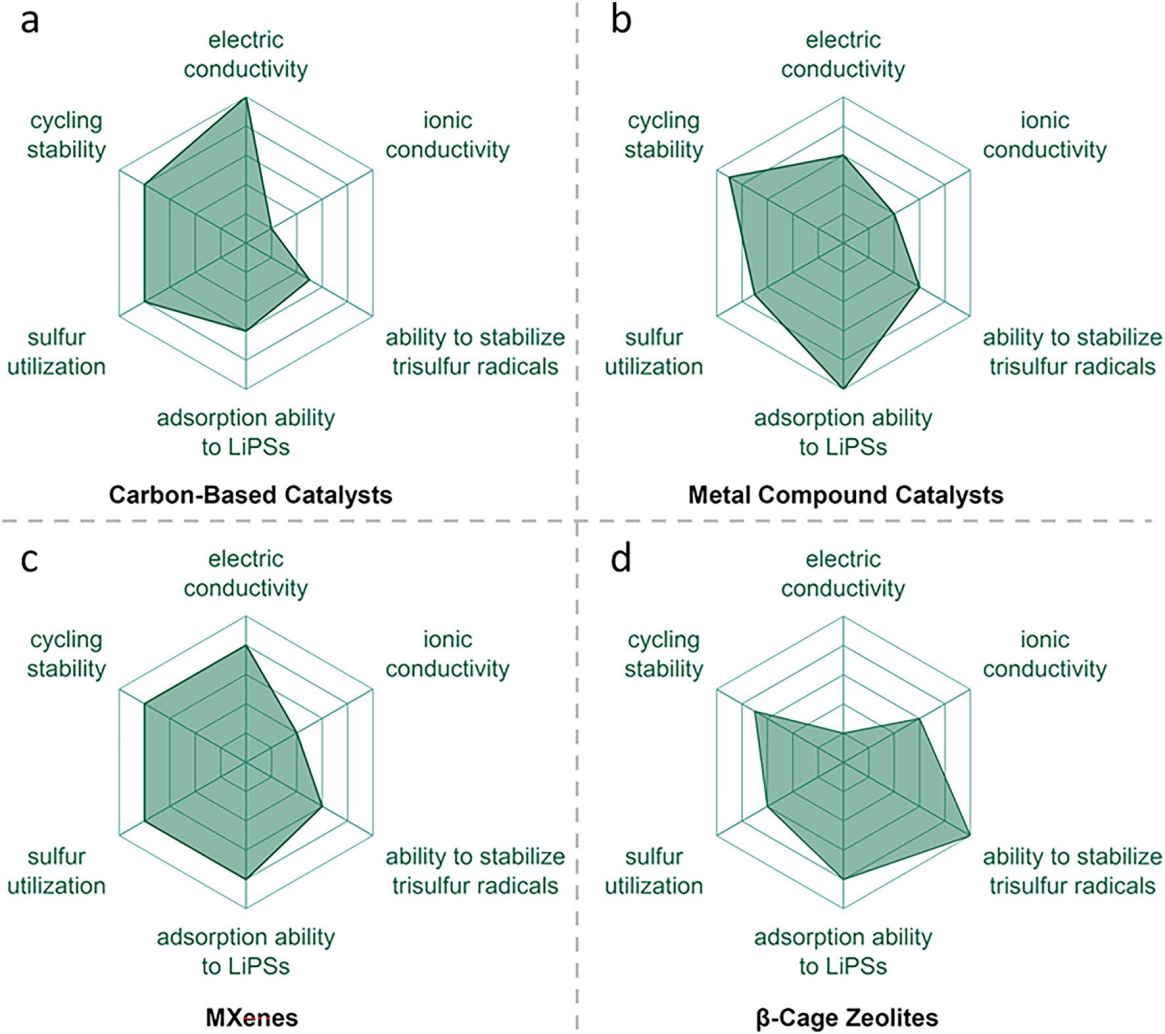
Radar map of heterogeneous electrocatalyst strategy with aspect of ionic conductivity, cycling stability, sulfur utilization, ability to stabilize ${\text{S}}_{3}^{\bullet-}$/${\text{LiS}}_{3}^{\bullet}$ radicals and metallic Li compatibility for **a** carbon-based catalysts, **b** metal compound catalysts, **c** MXenes, and **d** *β*-cage zeolites

Carbon-based catalysts, metal compound catalysts, MXenes, and sodium or *β*-cage zeolites serve as heterogeneous electrocatalysts with distinct advantages and limitations in stabilizing ${\text{S}}_{3}^{\bullet-}$/${\text{LiS}}_{3}^{\bullet}$ radicals and improving LSBs, as shown in Fig. 11. Carbon-based catalysts are notable for their excellent electronic conductivity and cycling stability but exhibit limited ability to stabilize ${\text{S}}_{3}^{\bullet-}$/${\text{LiS}}_{3}^{\bullet}$ radicals and adsorb LiPSs which hinders their effectiveness in suppressing the shuttle effect (Fig. 11a). To address this, surface modification with heteroatoms (such as nitrogen, sulfur, or metal atoms) can significantly enhance their chemical adsorption capability and catalytic activity for polysulfides, thereby improving overall performance. Metal compound catalysts, on the other hand, demonstrate outstanding adsorption ability for LiPSs (Fig. 11b), effectively suppressing the shuttle effect and enhancing cycling stability and sulfur utilization. However, their relatively low electronic and ionic conductivity poses a limitation. This can be mitigated by coupling metal compounds with highly conductive carbon materials or introducing defect structures (such as oxygen or sulfur vacancies) to improve their conductivity and catalytic activity.

MXenes, with their high electronic and moderate ionic conductivity, achieve a good balance in suppressing the shuttle effect and enhancing reaction kinetics, though their ability to stabilize ${\text{S}}_{3}^{\bullet-}$/${\text{LiS}}_{3}^{\bullet}$ radicals is slightly inferior (Fig. 11c). Surface modification or intercalation engineering (such as introducing functional groups or metal cations) could further enhance their ability to capture and stabilize ${\text{S}}_{3}^{\bullet-}$/${\text{LiS}}_{3}^{\bullet}$ radicals. Sodium or *β*-cage zeolites excel in ionic conductivity, strong LiPSs adsorption, and ${\text{S}}_{3}^{\bullet-}$/${\text{LiS}}_{3}^{\bullet}$ radicals’ stabilization (Fig. 11d), making them a promising candidate for suppressing the shuttle effect and promoting sulfur conversion. However, their limited electronic conductivity restricts their application under high-rate charge/discharge conditions. This can be addressed by compositing zeolites with conductive materials or tuning their pore structures (such as introducing metal ions to enhance electron transfer).

Thus, optimizing different catalysts through strategies such as surface modification, introducing heterogeneous structures, and functional design is essential to balance their performance in stabilizing ${\text{S}}_{3}^{\bullet-}$/${\text{LiS}}_{3}^{\bullet}$ radicals, adsorbing polysulfides, and enhancing overall electrochemical performance. These modifications and combination strategies provide crucial directions for the development of more efficient and stable catalysts for LSBs.

## Summaries and Perspectives

${\text{S}}_{3}^{\bullet-}$/${\text{LiS}}_{3}^{\bullet}$ radicals serve as essential intermediates in LSBs, facilitating sulfur conversion reactions, regulating Li_2_S deposition, and mitigating key challenges such as shuttle effects and electrode passivation. Below, we summarize several key issues discussed in this review that are central to the development of sulfur radicals in LSBs.

i. Theoretical calculations play an increasingly important role in understanding the formation and transformation mechanisms of ${\text{S}}_{3}^{\bullet-}$/${\text{LiS}}_{3}^{\bullet}$ radicals. First-principles calculations provide molecular-level insights into the reactions of ${\text{S}}_{3}^{\bullet-}$/${\text{LiS}}_{3}^{\bullet}$ radicals within the electrodes and electrolytes, guiding the design of electrolyte and catalyst materials to enhance battery performance. By integrating AIMD and classical dynamics simulations based on reactive force field (ReaxFF), the influence of electrolytes and catalysts on the stability of ${\text{S}}_{3}^{\bullet-}$/${\text{LiS}}_{3}^{\bullet}$ radicals and polysulfide shuttle behavior can be systematically studied, offering valuable theoretical support for electrolyte optimization. Additionally, theoretical calculations provide essential guidance for spectroscopic analysis, revealing the impact of electrolyte systems on the spectroscopic features of ${\text{S}}_{3}^{\bullet-}$/${\text{LiS}}_{3}^{\bullet}$ radicals, thereby facilitating the precise design of electrolytes and catalysts to improve the stability and energy efficiency of LSBs.
ii. The generation and catalytic behavior of ${\text{S}}_{3}^{\bullet-}$/${\text{LiS}}_{3}^{\bullet}$ radicals can be effectively monitored using advanced *in situ* characterization techniques, such as ESR, UV–Vis, Raman spectroscopy, and synchrotron XAS. These techniques offer critical insights into the stability, kinetics/dynamics, and electronic structure of ${\text{S}}_{3}^{\bullet-}$/${\text{LiS}}_{3}^{\bullet}$ radicals. However, challenges persist due to radicals’ transient nature, low concentration in traditional ether-based electrolytes, and the photosensitivity of their formation reactions. The introduction of radical trapping agents, such as nitrones and pyridinium cations, has significantly enhanced the stability of ${\text{S}}_{3}^{\bullet-}$/${\text{LiS}}_{3}^{\bullet}$ radicals and amplified spectral signals, enabling clearer elucidation of their generation and transformation. Integrating complementary techniques, such as ESR with XAS or UV–Vis with Raman spectroscopy, provides a more comprehensive understanding of radicals behavior and reaction pathways. Combining radical traps with UV–Vis and Raman improves signal detection, while synchrotron-based XAS reveals molecular-level interactions. Looking ahead, time-resolved spectroscopy and ultrafast laser techniques can capture radical’ rapid dynamics, and novel trapping agents and, alongside advanced material designs, will optimize radical-mediated catalysis and accelerate the development of high-energy–density, long-cycle-life LSBs.
iii. *β*-cage zeolites, a type of lapis lazuli analog containing trisulfur radicals, hold great potential as sulfur hosts for improving the performance of LSBs. These materials can be easily synthesized through reactions with sulfur and possess unique structural characteristics, enabling dual functionalities: mediating the stabilization of ${\text{S}}_{3}^{\bullet-}$/${\text{LiS}}_{3}^{\bullet}$ radicals and providing catalytic adsorption capabilities. Surface engineering through heteroatom doping or defect introduction can significantly enhance their adsorption and catalytic performance toward LiPSs. Integrating *β*-cage zeolites with conductive networks like graphene or carbon nanotubes effectively overcomes their limited electronic conductivity. Meanwhile, exploring other zeolite structures, such as SAPO molecular sieves and other artificial zeolites, offers new opportunities for ${\text{S}}_{3}^{\bullet-}$/${\text{LiS}}_{3}^{\bullet}$ radicals assembly. SAPO zeolites, with their tunable acidic sites and adjustable framework structures, show promise in precisely controlling the pathways and stability of ${\text{S}}_{3}^{\bullet-}$/${\text{LiS}}_{3}^{\bullet}$ radical formation, potentially enhancing catalytic activity and polysulfide adsorption. By introducing diverse framework structures and functionalized designs, these artificial zeolites can broaden their applications in energy storage, becoming strong candidates for next-generation high-efficiency sulfur hosts.
iv. Although high-DN solvents are considered effective media for stabilizing ${\text{S}}_{3}^{\bullet-}$/${\text{LiS}}_{3}^{\bullet}$ radicals, their high reactivity and viscosity can lead to metallic Li anode corrosion and excessive electrolyte consumption, limiting the cycle life and stability of the battery. High-DN anion-supported electrolytes or organic/inorganic additives, which stabilize ${\text{S}}_{3}^{\bullet-}$/${\text{LiS}}_{3}^{\bullet}$ radicals through the synergistic effects of anions and cations, present a promising solution. However, the chemical and electrochemical stability of these additives must be carefully addressed to prevent long-term performance degradation. Future research should focus on designing anion-cation pairs that balance radical stabilization with metallic Li anode compatibility, leveraging computational chemistry to identify low-reactivity high-DN anions. Additionally, integrating high-DN additives into hybrid or solid-state electrolytes, combined with dynamic protective interfaces or artificial SEI layers, can effectively mitigate lithium corrosion. Advanced *in situ* and *in operando* characterization techniques will also be essential to reveal the interactions between high-DN components, polysulfides, and lithium surfaces in real time.
v. Metal compounds with vacancies or defects, heteroatom-doped carbon materials, MXenes, and other novel solid-state catalyst as heterogeneous catalysts, not only possess traditional adsorption catalysis functions but also exhibit significant capabilities in promoting the generation of ${\text{S}}_{3}^{\bullet-}$/${\text{LiS}}_{3}^{\bullet}$ radicals. These materials show immense potential in enhancing sulfur utilization, suppressing the shuttle effect, and accelerating reaction kinetics in LSBs. However, their exact mechanisms in stabilizing ${\text{S}}_{3}^{\bullet-}$/${\text{LiS}}_{3}^{\bullet}$ radicals and increasing their concentration remain unclear, requiring further investigation. Future research could employ advanced characterization techniques to explore their dynamic behavior during ${\text{S}}_{3}^{\bullet-}$/${\text{LiS}}_{3}^{\bullet}$ radical generation and transformation. Additionally, rational design strategies, including the introduction of vacancy defects, heteroatom doping, and integration with conductive materials, could further optimize catalyst performance. Evaluating their long-term stability under practical conditions, such as high sulfur loading and low electrolyte content, is essential to address issues like structural degradation and active site passivation.
vi. Machine learning (ML) technology has shown tremendous potential in material design and battery performance optimization. By constructing appropriate descriptors and performing high-throughput calculations, ML can efficiently identify key factors influencing the stability and formation of ${\text{S}}_{3}^{\bullet-}$/${\text{LiS}}_{3}^{\bullet}$ radicals and reveal beneficial cathode materials, catalytic environments, and electrolyte characteristics that promote ${\text{S}}_{3}^{\bullet-}$/${\text{LiS}}_{3}^{\bullet}$ radical formation. Notably, advancements in constructing potential energy surfaces have significantly reduced the cost of traditional computational methods, extending the scale of simulations from the microscopic to the macroscopic level, providing additional insights into the role of ${\text{S}}_{3}^{\bullet-}$/${\text{LiS}}_{3}^{\bullet}$ radicals in battery performance. Furthermore, deep learning models can delve into the complex relationships between material properties and ${\text{S}}_{3}^{\bullet-}$/${\text{LiS}}_{3}^{\bullet}$ radicals stability by mining multidimensional data, offering theoretical guidance for designing efficient electrolytes and catalysts.

In summary, future research should focus on optimizing the design of high-DN solvents and additives, as well as the development of advanced heterogeneous catalysts that can effectively stabilize and promote trisulfur radicals without compromising the stability of the metallic Li anode. Furthermore, integrating computational models and ML with advanced characterization techniques will be essential for elucidating the formation mechanisms and catalytic roles of ${\text{S}}_{3}^{\bullet-}$/${\text{LiS}}_{3}^{\bullet}$ radicals. These efforts will pave the way for designing more efficient and durable LSBs and potentially other metal − sulfur batteries, offering enhanced energy density, rate capability, longer cycle life, and improved overall performance.
